# A Cell Cycle and Nutritional Checkpoint Controlling Bacterial Surface Adhesion

**DOI:** 10.1371/journal.pgen.1004101

**Published:** 2014-01-23

**Authors:** Aretha Fiebig, Julien Herrou, Coralie Fumeaux, Sunish K. Radhakrishnan, Patrick H. Viollier, Sean Crosson

**Affiliations:** 1 Department of Biochemistry and Molecular Biology, University of Chicago, Chicago, Illinois, United States of America; 2 Department of Microbiology and Molecular Medicine, Institute of Genetics & Genomics in Geneva (iGE3), University of Geneva Medical School, Geneva, Switzerland; 3 Committee on Microbiology, University of Chicago, Chicago, Illinois, United States of America; Universidad de Sevilla, Spain

## Abstract

In natural environments, bacteria often adhere to surfaces where they form complex multicellular communities. Surface adherence is determined by the biochemical composition of the cell envelope. We describe a novel regulatory mechanism by which the bacterium, *Caulobacter crescentus*, integrates cell cycle and nutritional signals to control development of an adhesive envelope structure known as the holdfast. Specifically, we have discovered a 68-residue protein inhibitor of holdfast development (HfiA) that directly targets a conserved glycolipid glycosyltransferase required for holdfast production (HfsJ). Multiple cell cycle regulators associate with the *hfiA* and *hfsJ* promoters and control their expression, temporally constraining holdfast development to the late stages of G1. HfiA further functions as part of a ‘nutritional override’ system that decouples holdfast development from the cell cycle in response to nutritional cues. This control mechanism can limit surface adhesion in nutritionally sub-optimal environments without affecting cell cycle progression. We conclude that post-translational regulation of cell envelope enzymes by small proteins like HfiA may provide a general means to modulate the surface properties of bacterial cells.

## Author Summary

Bacteria predominantly exist within surface-attached communities that facilitate metabolic cooperation, sharing of genetic information, and protect cells against stress. The freshwater bacterium, *Caulobacter crescentus* elaborates an adhesive structure known as the holdfast, which enables surface attachment. We have discovered a novel mechanism that controls holdfast development in response to cell cycle and environmental cues. This regulatory mechanism involves a small protein inhibitor, HfiA, which targets a conserved holdfast synthesis enzyme and ensures that the holdfast is produced at the appropriate stage of cell development and under the appropriate environmental conditions. To our knowledge, the regulatory system we report here is unprecedented, and provides a mechanism for integrative control of bacterial cell adhesion in response to cell cycle and environmental signals.

## Introduction

The majority of bacteria in the biosphere exist within surface-attached communities [1]–[3] that facilitate metabolic cooperation, sharing of genetic information, and protect cells against stress (reviewed in [1]). Environmental signals including nutrient availability, pH, and ion concentrations influence surface community formation by modulating expression of adhesive cell envelope structures and extracellular polymers that determine surface attachment (reviewed in [4]). The Gram negative bacterium, *Caulobacter crescentus*, thrives in dilute freshwater ecosystems and has the ability to permanently attach [5], [6] to a chemically diverse range of surfaces [7]–[10] via a polysaccharide-rich, polar organelle known as the holdfast [9], [11]–[13]. As organic polymers and ions concentrate on material surfaces in aquatic environments [14], surface attachment likely provides *C. crescentus* a nutritional advantage. Given that holdfast surface attachment is permanent, *C. crescentus* should exhibit tight control over holdfast development to ensure that cells do not become perpetual residents of a poor environment. In this study, we have sought to elucidate the molecular regulatory determinants of holdfast development in *C. crescentus*.

**Figure 1:**
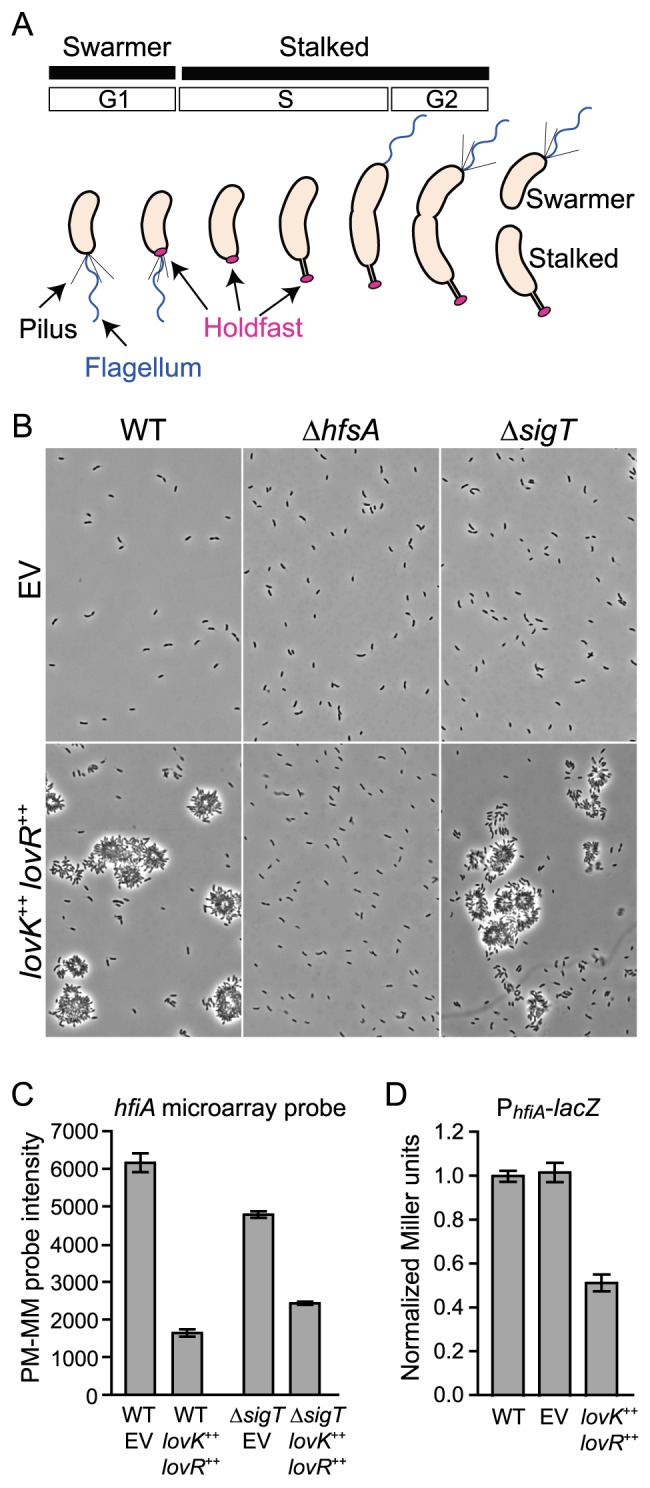
*lovK-lovR*–enhanced adhesion and transcriptional repression of *CC_0817 (hfiA)*. **A.** *C. crescentus* cell cycle yields two cell types: swarmer and stalked. Development of polar structures including pili (black), flagellum (blue) and the adhesive holdfast (pink) is cell-cycle regulated. **B.** Phase contrast micrographs of wild-type (WT), Δ*hfsA* or Δ*sigT* cells bearing either empty plasmids (EV) or *lovK* and *lovR* overexpression plasmids (*lovK* ^++^, *lovR* ^++^) grown in M2XV medium. **C.** Affymetrix microarray expression values (perfect match minus mismatch probes: PM-MM) indicating transcript level for *hfiA* in log phase cells grown in M2XV. Bars represent mean ± range of two independent cultures. *hfiA* transcript is significantly lower in *lovK-lovR* overexpression strains compared to isogenic empty vector controls (ANOVA followed by Tukey's post test; p<0.001). **D.** β-galactosidase activity from a P*_hfiA_*-*lacZ* transcriptional fusion (pRKlac290-P*_hfiA_*) in wild-type (WT), vector control (EV), and *lovK*–*lovR* overexpression strains grown in M2XV. Bars represent mean ± s.e.m. of 11 independent cultures assayed on 4 different days. P*_hfiA_*-*lacZ* activity in the *lovK*-*lovR* overexpression strain is significantly lower than WT and vector control strains (ANOVA followed by Tukey's post test; p<0.001).

Elaboration of the holdfast adhesin in *C. crescentus* is cell-cycle-regulated, though it is not requisite for cell-cycle progression [8], [15]–[17]. The cell cycle yields two cell types that are physiologically, morphologically and functionally distinct (Figure 1A). The flagellated and motile swarmer cell provides this species a means for dispersal; this cell type is arrested in G1 and incapable of replication. In order to initiate growth and replication, the swarmer relinquishes motility and differentiates into a stalked cell. The stalked cell, specialized for nutrient uptake, grows and divides asymmetrically to generate a new swarmer cell upon division [8], [18]. Development of the holdfast at the cell surface is temporally restricted to the late swarmer cell stage, where it emerges at the nascent stalked cell pole ([15], [17], Figure 1A). However, the timing of holdfast emergence within this developmental window can be hastened at the post-translational level by physical contact of the flagellum with surfaces [19]. Once constructed, the holdfast is a permanent feature of the cell surface that is not shed or reassimilated. Premature holdfast development at the nascent swarmer pole prior to cell division would hinder dispersal of newborn swarmer cells. Thus cell-cycle control of holdfast biogenesis helps to ensure appropriate cell dispersal.

We have previously observed that a two-component regulatory system composed of the soluble sensor histidine kinase, LovK, and the single domain receiver, LovR, regulates the *Caulobacter* general stress response [20] and modulates cell adhesion [21]. We sought to understand the mechanism of adhesion control and have discovered a novel inhibitor of holdfast development, *hfiA*, that is regulated downstream of *lovK-lovR*. A forward genetic screen for HfiA-insensitive mutants identified suppressing mutations in a glycosyltransferase gene required for holdfast development, which we name *hfsJ*. We demonstrate a physical interaction between HfiA and HfsJ, and that suppressing mutations in HfsJ attenuate the HfsJ-HfiA interaction. These results support a model in which HfiA inhibits holdfast development via direct interaction with an enzyme required for holdfast biosynthesis.

Expression of *hfiA* is temporally regulated across the cell cycle, and is lowest during the period when the holdfast is elaborated at the cell surface. Multiple *Caulobacter* developmental regulators, CtrA, GcrA and StaR, physically occupy and control transcription from the *hfiA* promoter. The coordinate action of these regulators induces *hfiA* at the end of G1, thus restricting holdfast formation to the swarmer cell. However, not every cell makes a holdfast; the probability of holdfast emergence at the single cell level depends on the nutritional composition of the growth medium and is inversely correlated with *hfiA* expression. Our data thus support a model in which holdfast development is controlled by cell cycle and nutritional input signals that are integrated at the promoter of *hfiA*. As a negative regulator of an enzyme required for holdfast production, HfiA functions as a checkpoint protein that ensures holdfast development occurs within the appropriate cell cycle window and nutritional conditions.

## Results

### *lovK-lovR*-enhanced adhesion requires holdfast synthesis

We previously observed that coordinate overexpression of *lovK* and *lovR* increases cell-cell adhesion, and deletion of *lovK* or *lovR* reduces adhesion [21]. To understand the genetic basis of this adhesion phenotype, we first tested if the holdfast is required for *lovK-lovR*-enhanced adhesion. We overexpressed *lovK* and *lovR* in a strain lacking *hfsA*, a gene required for holdfast synthesis [13]. In a wild-type background, overexpression of *lovK* and *lovR* results in large cell aggregates that are readily visible in the culture (Figure 1B) and accumulate in a ring along the culture tube wall (Figure S1). Strains lacking *hfsA* do not exhibit enhanced cell-cell adhesion or tube ring formation upon *lovK-lovR* overexpression. Thus, holdfast is required for the *lovK-lovR*-enhanced adhesion phenotype.

We recently discovered that *lovK* and *lovR* are potent negative regulators of the general stress response (GSR) sigma factor σ^T^ and, concordantly, function as negative regulators of cell survival during osmotic and oxidative stress [20]. As σ^T^ controls expression of a number of genes involved in cell envelope function [20], [22], we hypothesized that *lovK*-*lovR* affects adhesion via σ^T^-dependent modulation of the cell envelope. Our hypothesis predicts that cells lacking *sigT* should be hyper-adhesive. However, a Δ*sigT* null strain is not hyper-adhesive. Moreover, coordinate overexpression of *lovK*-*lovR* in Δ*sigT* background results in an equivalent cell adhesion phenotype as a strain expressing *lovK-lovR* in a wild-type background (Figures 1B & S1). These data demonstrate that *lovK-lovR* modulates adhesion independent of *sigT*, via a mechanism that requires holdfast development.

### LovK-LovR represses transcription of *cc_0817* (*hfiA*), a novel inhibitor of holdfast development

We next sought to identify specific adhesion effector(s) regulated by LovK-LovR, independent of σ^T^. We measured change in global transcript abundance upon *lovK-lovR* overexpression in *ΔsigT* and in wild-type genetic backgrounds. Only one transcript, *cc_0817* (*ccna_00860*), exhibited differential steady-state levels in both experiments; showing a 2–3-fold reduction (Figure 1C). A *lacZ* transcriptional fusion to the *cc_0817* promoter confirmed that overexpression of *lovK*-*lovR* represses *cc_0817* transcription (Figure 1D). *cc_0817* mRNA is among the top 10% of transcripts in the *C. crescentus* cell in terms of abundance, based on analysis of wild-type expression array data [20]. Thus modest fold changes in transcript abundance reflect large changes in absolute number of transcripts.

Our finding that *lovK-lovR* overexpression results in decreased transcription of *cc_0817* and increased holdfast-dependent adhesion suggested that *cc_0817* functions downstream of *lovK-lovR* as an inhibitor of holdfast development. To test this hypothesis, we assayed the effect of *cc_0817* deletion and overexpression on holdfast development. Holdfast development was monitored by incubating cells with fluorescently-labeled wheat germ agglutinin (WGA-Alexa595), a lectin that binds N-acetylglucosamine and marks holdfast at the cell surface [9].

**Figure 2:**
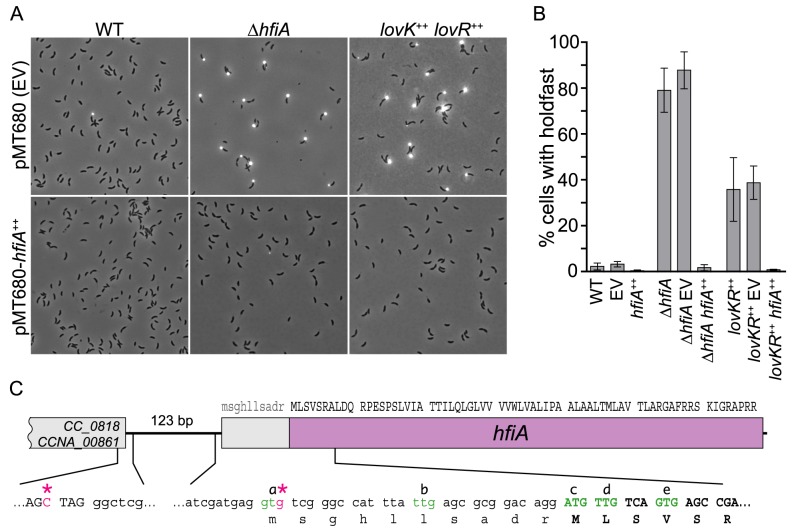
CC_0817 functions as a *h*old*f*ast *i*nhibitor (HfiA). **A.** Representative micrographs of cells grown in M2XV medium and incubated with WGA-Alexa594 to visualize holdfast. Strains carry either empty vector (EV) control plasmid (top row) or a xylose inducible *hfiA* overexpression plasmid (bottom row). **B.** Quantification of cells displaying holdfast. Bars indicate mean ± s.d. of at least three independent samples of each genotype. At least 300 cells were counted in each sample. **C.** *hfiA* locus with DNA sequences of selected regions. Triplets indicate predicted coding sequence. Transcription start sites mapped using 5′ RACE are marked with pink asterisks (*). NTG codons that could function as translation start sites are green and annotated a-e. Reannotated coding sequence is uppercase.

In minimal defined medium with xylose as the carbon source, about 3% of wild-type cells display a holdfast (Figure 2A&B). Overexpression of *cc_0817* reduces the fraction of cells with a visible holdfast to near zero. *lovK-lovR* overexpression increases the fraction of cells with a holdfast ∼10-fold and *cc_0817* overexpression in this background (*lovK-lovR* ^++^ *cc_0817* ^++^) attenuates this effect (Figure 2A&B). Conversely, deletion of *cc_0817* results in elaboration of a holdfast on nearly every cell (Figure 2A&B). Expression of *cc_0817* from the ectopic *xylX* locus in a Δ*cc_0817* null background restores wild-type holdfast levels. These data support a model in which *cc_0817* functions to inhibit holdfast development. We have named this gene *h*old*f*ast *i*nhibitor A, *hfiA*.

### *hfiA* encodes a small novel protein

*hfiA* was annotated as a 78 aa hypothetical protein [23]; the central portion of the putative protein contains a hydrophobic stretch of 35 amino acids. A search of the Pfam and Conserved Domain Databases with the primary sequence of HfiA revealed no conserved domains. Given the small predicted size of *hfiA* and the lack of functional clues in the sequence, we sought to validate the prediction that *hfiA* is translated into a protein, and to define the length of the predicted open reading frame.

We identified two *hfiA* transcriptional starts by 5′ RACE. Seventy-five percent of the sequenced RACE products started at the third position of the predicted *hfiA* translational start codon and twenty-five percent started 126 bp upstream of the predicted translational start (Figure 2C). These data suggested the *hfiA* start codon was annotated incorrectly. The first 14 codons of the annotated *hfiA* coding sequence include 4 additional NTG codons (marked *b*, *c*, *d*, and *e* in Figure 2C) that could potentially function as translation start sites. To test if putative codons *a* or *b* function as translation start sites, we expressed from the *hfiA* promoter a translational fusion between the first 7 predicted *hfiA* codons and *lacZ*. We engineered a second translational fusion that also included codons c, d and e fused to *lacZ*. Only the second fusion yielded β-galactosidase activity above background (Figure S2), demonstrating that *hfiA* is translated and that translation initiates from codons *c*, *d* or *e*.

To identify the site of translation initiation we replaced the wild-type *hfiA* allele with mutant alleles in which codons *c*, *d* or *e* were mutated away from NTG, and thus could no longer function as translation start sites. We predicted that loss of the translation start would phenocopy the hyper-holdfast phenotype of the Δ*hfiA* null strain. However, no single codon mutant exhibited a null phenotype (Figure S2), suggesting *hfiA* translation can initiate at multiple sites. Furthermore, no double codon mutant exhibited a full hyper-holdfast phenotype. Only the strain bearing mutations in all three putative start codons (*c*, *d* and *e*) phenocopied the Δ*hfiA* null strain (Figure S2). Thus all three of these codons can likely function as sites of translation initiation, resulting in synthesis of 65–68 amino acid proteins (∼7 kDa). We have reannotated *hfiA* to reflect initiation at the ATG that was originally predicted to be codon 11.

### A forward genetic screen identifies HfiA suppressor mutations

**Figure 3:**
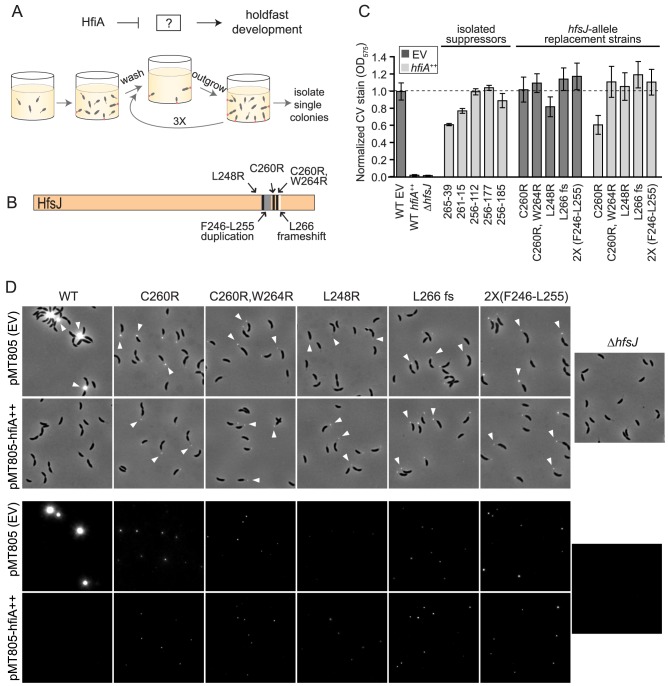
HfiA suppressor screen identifies a novel holdfast synthesis gene, *hfsJ*. **A.** Schematic of the expected target of a forward genetic screen for HfiA suppressors, and a strategy to enrich for mutants that are insensitive to *hfiA* overexpression (see Materials and Methods for details). **B.** Summary of *hfsJ* mutations in the 5 strains that suppress the *hfiA* overexpression phenotype. Black lines, non-synonymous SNPs; grey, duplicated region; and white, out-of-frame deletion. **C.** Surface attachment after growth in polystyrene plates measured by crystal violet staining of attached cells for original suppressor strains, and strains bearing targeted mutations in *hfsJ*. All strains carry either the empty vector (pMT805; dark grey bars) or the *hfiA* overexpression plasmid (light grey bars). Data represents mean ± s.d.; n = 16. Data were collected over 4 days and normalized to the wild-type empty vector control on each day. **D.** Strains with directed mutations in the *hfsJ* locus elaborate a holdfast in the presence of *hfiA* overexpression. WGA-Alexa595 lectin staining of cells grown in PYE supplemented with 0.15% xylose to induce expression. Holdfast (white arrowheads) on wild-type and *hfsJ* mutants are shown for strains carrying the empty plasmid (EV) or *hfiA* overexpression plasmid. Top panel: phase contrast and fluorescence combined. Bottom panel: fluorescence signal alone. Intensity is scaled identically across all images in each panel.

We hypothesized that the small protein, HfiA, functions to directly inhibit a protein required for holdfast development. To test this hypothesis, we designed an unbiased genetic screen to identify adhesive mutants that continue to produce holdfast when *hfiA* is overexpressed (Figure 3A). Several classes of adhesive mutants emerged from this screen: *a)* Mutants in which *hfiA* overexpression was disrupted by lesions in the xylose-inducible promoter, in the *hfiA* coding sequence (Table S1 & Figure S3), or in the xylose transport system; *b)* Mutants with increased surface adhesion but not increased holdfast including lesions in the gene encoding the S-layer protein or in genes involved in synthesis of lipopolysaccharide (LPS), which attaches the S-layer protein to the cell [24]–[26]; *c)* Mutants with an elevated number of holdfast-bearing cells in the presence of an intact *hfiA* overexpression system. We initially isolated two independent *hfiA*-suppressor strains, 256-39 and 261-15, with strongly enhanced surface adhesion (Figure 3C) and a high fraction of cells bearing a holdfast.

Whole genome sequencing of these suppressors revealed multiple mutations relative to the wild-type parent (Table S1). While each suppressor strain bore unique mutations, both 256-39 and 261-15 shared non-synonymous polymorphisms in gene *cc_0095* (*ccna_00094*) that resulted in a C260R substitution and C260R,W264R substitutions, respectively.

We conducted additional enrichment screens and identified three other independent HfiA-supressors (256-112, 256-177 and 256-185) that exhibit near wild-type surface adhesion when *hfiA* is overexpressed (Figure 3C). Targeted sequencing of the *cc_0095* locus in these strains revealed that each harbor mutations in the 3′ end of this gene which result in the following coding changes: L248R, a frame-shift after L266 and a duplication of F246-R254 respectively (Table S1, Figure 3B). The independent isolation of five unique lesions in the same region of *cc_0095* strongly implicated this gene in *hfiA*-mediated control of holdfast development.

CC_0095 is annotated as a UDP-N-acetyl-D-mannosaminuronic acid transferase and is related to *E. coli* WecG (29% identity/45% similarity) and *Bacillus subtilis* TagA (27% identity/46% similarity) glycosyltransferases. This protein is strongly classified as a WecG/TagA–family glycosyltransferase in the Conserved Domain Database (E-value<e^−84^) [27], though the glyco-substrates are difficult to predict from primary sequence. This family of enzymes is widely distributed in Gram-negative and Gram-positive bacteria. Within the *Caulobacterales*, all sequenced species that encode the holdfast synthesis gene cluster, *hfsEFGHCBAD*, also encode proteins that are 50–80% identical to CC_0095.

### *cc_0095* (*hfsJ*) is required for holdfast development

WecG/TagA–family enzymes catalyze the transfer of an activated nucleotide sugar to a glycosylated membrane phospholipid, undecaprenyl pyrophosphate (Und-PP) [27]. Execution of this chemistry is a critical early step in the biosynthesis of extracellular sugar polymers including the holdfast material [12]. To test if *cc_0095* functions in holdfast development, we generated a strain carrying an in-frame deletion of this gene. Strains lacking *cc_0095* do not develop holdfast (Figure 3D) and are completely defective in surface adhesion (Figure 3C) consistent with the defective biofilm phenotype reported for a *cc_0095* transposon insertion mutant [28]. Expression of *cc_0095* from an ectopic locus restores holdfast synthesis and surface adhesion to the null mutant (Figure S4). Neither *E. coli wecG* nor *B. subtilis tagA* complements the Δ*cc_0095* adhesion or holdfast defects, although expression of *E. coli* WecG alters *C. crescentus* morphology resulting in cells that are longer, thinner, and no longer curved (Figure S4). Genes required for *h*old*f*ast *s*ynthesis have been assigned the names *hfsA* through *hfsI* [12]. We have named gene *cc_0095*, *hfsJ*.

We note that *hfsJ* prediction is 10 codons shorter in the *C. crescentus* NA1000 (CCNA_00094) genome annotation compared to strain CB15 (CC_0095) annotation. To experimentally define the start codon, we generated strains in which each predicted start codon was mutated. Only mutation of the predicted start annotated in NA1000 phenocopied the null, supporting the annotation of the shorter open reading frame (Figure S4); this was used as the frame of reference for numbering the position of *hfsJ* mutations.

A fluorescent protein fusion, HfsJ-venus, expressed from the native *hfsJ* promoter complements the holdfast null Δ*hfsJ* phenotype (Figure S4). In contrast to holdfast export and anchoring proteins, which are localized to the stalked pole [11], [29], HfsJ-venus is distributed throughout the cell (Figure S4). Western blot analysis on this strain with antibodies to GFP/venus indicates that the HfsJ-venus fusion is not cleaved; no degradation products were detected (Figure S4). Thus the fluorescence signal observed throughout the cell reflects the distribution of HfsJ-venus. Moreover, HfsJ-venus was detected only in the pellet fraction but not the soluble fraction of the cell lysate (Figure S4), supporting a model in which this protein is membrane associated. We cannot rule out the possibility that the fluorescent tag either alters the localization of HfsJ, or stabilizes it so that a localization site becomes saturated. The chemistry that HfsJ is predicted to execute (modification of an inner membrane carrier glycolipid) occurs in the cytoplasm. Given the rapid two-dimensional diffusion of lipids in the inner membrane, such a lipid-modifying enzyme need not be spatially restricted to produce a product that is utilized by the holdfast synthesis and export machinery located at the nascent stalked cell pole.

### Lesions near the C-terminus of HfsJ suppress holdfast inhibition by HfiA

To test if the *hfsJ* lesions identified in our genetic screen specifically suppress the holdfast inhibition function of *hfiA*, we constructed strains in which we replaced wild-type *hfsJ* with each of the mutant alleles. These *hfsJ*-mutant strains were transformed with either empty plasmid or the *hfiA* overexpression plasmid and assayed for surface adhesion and visible holdfast.

Each of the *hfsJ*-mutant strains exhibits surface adhesion in the presence of the empty control plasmid (Figure 3C, dark bars). Thus, the mutations in *hfsJ* do not compromise bulk adhesion. However, the holdfast in these strains do not stain as intensely as wild-type (Figure 3D) suggesting they may be smaller than wild type. While overexpression of *hfiA* nearly abolishes surface adhesion and holdfast development in wild-type cells (Figure 3C&D), the *hfsJ*-mutant strains are largely insensitive to the effect of *hfiA* overexpression on surface adhesion and holdfast formation (Figure 3C&D).

### HfiA and HfsJ directly interact *in vitro* and *in vivo*

**Figure 4:**
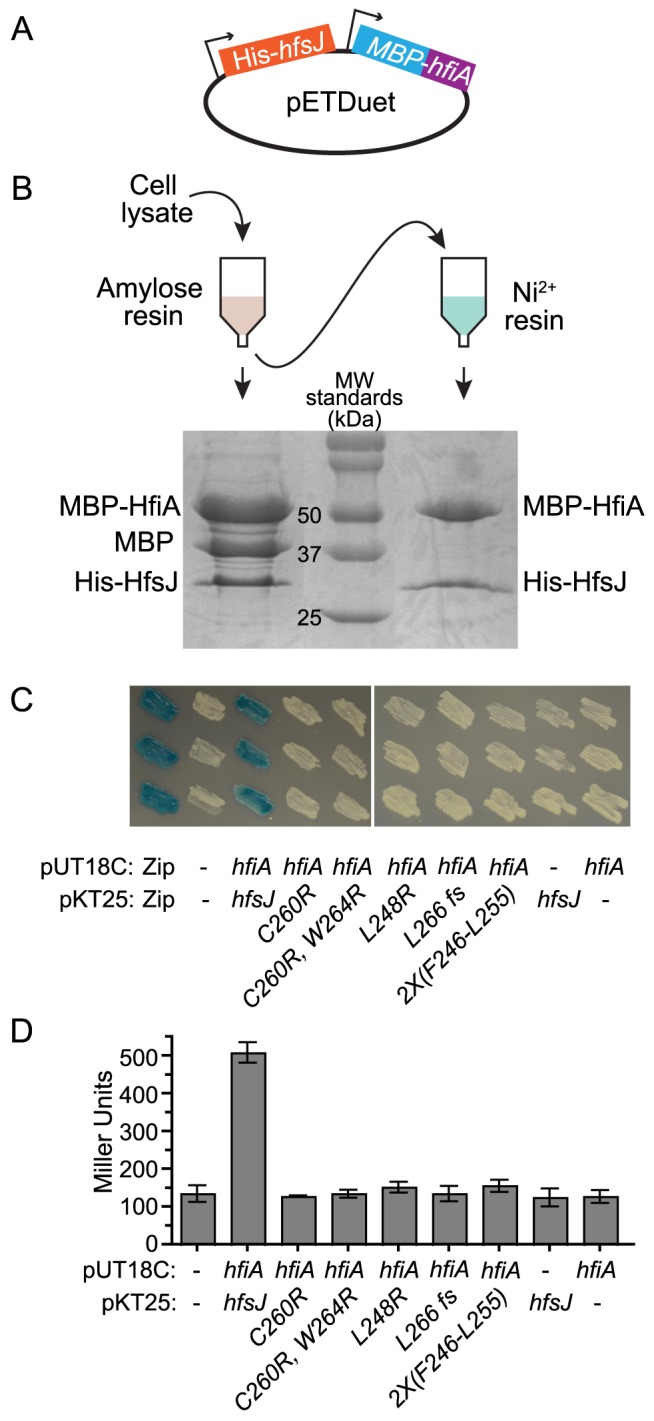
HfiA and HfsJ directly interact in vitro and in vivo. **A.** pETDuet plasmid used to co-express His6-*hfsJ* and MBP-*hfiA* from T7 promoters. **B.** Co-affinity purification scheme and Coomassie stained SDS-PAGE gel of proteins purified first with amylose resin followed by affinity purification with Ni^2+^ resin. **C.** Bacterial two-hybrid assay. *E. coli* cells bearing plasmids with fusions to either the T18c or T25 domains of adenylate cyclase were grown on medium containing X-gal. Interaction between the fusion proteins results in expression of *lacZ* and conversion of X-gal to yield blue colonies. T18c was fused to either a positive control (Zip) or *hfiA*. T25 was fused to a positive control (Zip), *hfsJ* or *hfsJ* mutant alleles. Dashes indicate empty vector controls. Three independent colonies of each combination are shown. **D.** β-galactosidase activity from strains expressing the fusions in C. Bars represent mean ± s.d. (n = 3). Only the strain with fusions to wild-type alleles of *hfiA* and *hfsJ* yielded β-galactosidase activity significantly (p<0.01) different from the control strain with split domains lacking fusions (one-way ANOVA followed by Dunnett's multiple comparison post-test).

To test if HfiA and HfsJ physically interact, we assayed whether the proteins co-purify by serial affinity chromatography. We cloned *hfiA* and *hfsJ* into a tandem *E. coli* expression plasmid, with N-terminal maltose binding protein (MBP) and His_6_ affinity tags, respectively (Figure 4A). MBP-HfiA (51 kDa) and His_6_-HfsJ (33 kDa) co-eluted from amylose affinity resin (Figure 4B). We observed an additional band, the size of the MBP tag alone (42 kDa), suggesting that the MBP-HfiA fusion is partially unstable. Eluate from the amylose resin was then bound to Ni^2+^ resin. Only two proteins of sizes corresponding to His_6_-HfsJ and MBP-HfiA co-eluted from the Ni^2+^ resin (Figure 4B). We confirmed the identities of these proteins as His_6_-HfsJ and MBP-HfiA by mass spectrometry. As a control, we confirmed that MBP-HfiA does not bind to Ni^2+^ resin, nor does His_6_-HfsJ bind to amylose resin, nor is the interaction mediated by the MBP domain (Figure S5). Together these data provide strong support for a direct physical interaction between HfsJ and HfiA.

To provide additional support for a physical interaction between HfiA and HfsJ, we preformed a bacterial two-hybrid assay [30]. Co-expression of T25-*hfsJ* and T18-*hfiA* fusions results in blue colonies when grown on medium containing X-gal (Figure 4C), and significant β-galactosidase activity when grown in liquid (Figure 4D), demonstrating that HfsJ and HfiA interact and bring together the split T25/T18 adenylyl cyclase domains. Reconstitution of adenylyl cyclase activity required both fusions; neither fusion alone was sufficient. Importantly, none of the HfsJ mutant alleles interact with HfiA sufficiently to yield a positive result in this assay (Figure 4C&D).

These data support a model in which HfiA functions to inhibit holdfast development through direct interaction with HfsJ, a putative glycosyltransferase required for holdfast development.

### *hfiA* expression is cell-cycle regulated and inversely correlated with holdfast development

**Figure 5:**
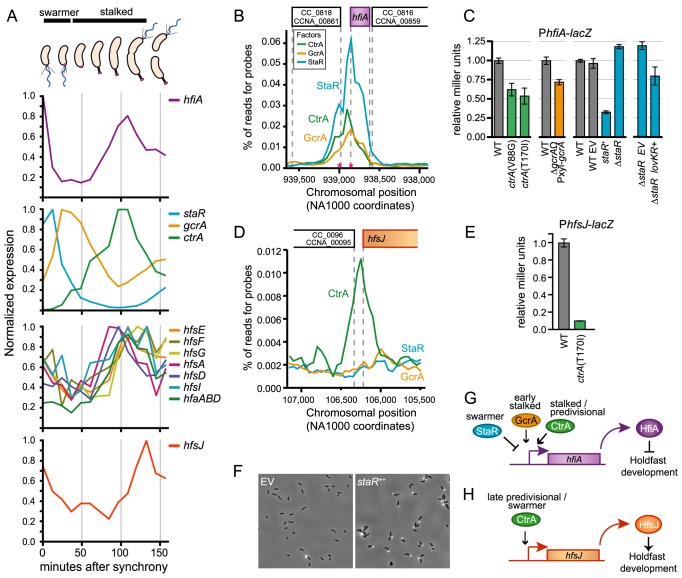
Transcription of *hfiA* is coordinately controlled by multiple regulatory proteins. **A.** Cell cycle-dependent transcriptional regulation of genes involved in holdfast development. Data were extracted from global transcriptional profiling experiments [33] and normalized to maximal expression for each gene. Approximate developmental stage at each time point is shown at top. **B.** The core cell cycle regulators StaR, CtrA and GcrA occupy the *hfiA* promoter. ChIP-seq data of the *hfiA* locus for each regulator. Chromosomal position of DNA pulled down with each of the regulators is indicated below; the gene context is indicated above. Pink asterisks indicated mapped transcriptional start sites for *hfiA*. **C.** β-galactosidase activity from a P*_hfiA_*-*lacZ* transcriptional fusion (pRKlac290-P*_hfiA_*) assayed in strains bearing temperature sensitive alleles of *ctrA* outgrown in PYE at 37°C for 4 hours, in *gcrA* depletions strains grown in the absence of xylose (PYE+0.15% glucose) for 6 hours, and in strains in which *staR* was deleted or overexpressed from the P*_xyl_* promoter (PYE+0.15% xylose). In each experiment, Miller units were normalized to WT grown in parallel conditions. Data represent mean ± s.d. of 6 independent cultures assayed on two different days. **D.** CtrA, but not GcrA or StaR, occupies the *hfsJ* promoter. ChIP-seq data for the *hfsJ* locus annotated as in B. **E.** CtrA activates transcription from the *hfsJ* promoter. β-galactosidase activity from a P*_hfsJ_*-*lacZ* transcriptional fusion in wild-type and *ctrA* ^ts^ strains at restrictive temperature (37°C for 3 hours). Data represent mean ± s.d. of 4 independent cultures assayed on two different days. **F.** WGA-Alexa594 staining of holdfast in wild-type empty vector (EV) control strain or a *staR* ^++^ overexpression strain grown in M2X. **G.** Model of the *hfiA* regulatory activities of CtrA, GcrA and StaR. **H.** Model of the *hfsJ* regulatory activities of CtrA.

Holdfast development is temporally regulated across the cell cycle [8], [15], [17], [19]: the holdfast is elaborated at the flagellated pole of the swarmer cell, before or during the swarmer-to-stalked cell transition (Figure 1A). Profiles of *C. crescentus* gene expression throughout the cell cycle reveal that transcription of the holdfast inhibitor *hfiA* is cell-cycle regulated, with a minimum at the period of holdfast development (Figure 5A), [31]–[33]). These results are consistent with a model in which cell-cycle regulation of *hifA* expression determines the developmental window for holdfast biogenesis.

### Holdfast development is connected to the cell cycle control network via *hfiA*

*C. crescentus* cell cycle progression is controlled via dynamic interplay between a number of developmental regulatory proteins (reviewed by [34]–[36]). Three known developmental regulators, CtrA, GcrA, and StaR directly control *hfiA* expression. These proteins are introduced briefly here: *a)* CtrA, an essential response regulator with a DNA-binding output domain [37], is a ‘master developmental regulator’ that directly or indirectly controls transcription of ∼25% of the *C. crescentus* cell cycle regulated genes [32], [37]. *b)* GcrA is critical developmental regulator required for efficient growth that forms a feedback control loop with CtrA [38], [39]. *c)* StaR is a non-essential developmental regulator that controls stalk biogenesis [40]. Transcription of these genes is temporally regulated across the cell cycle (Figure 5A; [31]–[33]).

StaR, CtrA and GcrA physically occupy the chromosomal region immediately upstream of *hfiA* (Figure 5B; Tables S2, S3, S4, S5, and [41]). To test whether these proteins affect *hfiA* expression, we assayed transcription from a P*_hfiA_*-*lacZ* transcriptional fusion in *ctrA*, *gcrA*, or *staR* mutant backgrounds. Cells lacking *ctrA* and *gcrA* are severely compromised and have developmental defects thus, we used temperature sensitive alleles [37], [42] or depletion strains [38] to evaluate the effects of protein loss on *hfiA* transcription. At restrictive temperatures, strains bearing *ctrA ts* alleles have 2-fold less P*_hfiA_*-*lacZ* activity than wild type; P*_hfiA_*-*lacZ* activity is reduced ∼25% upon GcrA depletion (Figure 5C). We conclude that CtrA and GcrA are transcriptional activators of *hfiA* (Figure 5G). Overexpression of *staR* from a xylose-inducible promoter reduced P*_hfiA_*-*lacZ* activity by ∼70%, deletion of *staR* enhanced P*_hfiA_*-*lacZ* activity by ∼20% (Figure 5C). These data provide evidence that StaR represses *hfiA* transcription. We note that our experiments with unsynchronized populations will mask the amplitude of temporally-restricted transcriptional change. Indeed, endogenous StaR is expected to affect only a subset of cells in the population at any given time. Conversely, overexpression of *staR* from an inducible promoter affects P*_hfiA_* in all of the cells at any given time.

Like many genes involved in holdfast biogenesis, transcription of *hfsJ* is also cell cycle regulated (Figure 5A; [31]–[33]). Sequences corresponding to the *hfsJ* locus are enriched by immunoprecipitation of CtrA, but not GcrA or StaR (Figure 5D). Transcription from the *hfsJ* promoter is diminished in a *ctrA* temperature sensitive (*ctrA^ts^*) mutant (Figure 5E); we conclude that CtrA is a direct activator of *hfsJ* transcription (Figure 5H). We identified CtrA binding sites upstream of both *hfiA* and *hfsJ* (CTCttaaAGCTTTCtaaaCCT, 92 bp, p = 1.0e-04 and ATActtaGCGGGATttaaCCA, 66 bp, p = 6.3e-07 respectively).

We next asked whether the activity StaR on *hfiA* transcription affects holdfast development. Both *ctrA* and *gcrA* are essential, and mutant strains have pleiotropic defects that confound assessment of holdfast development. The function of StaR was initially investigated in a holdfast deficient genetic background; thus no holdfast phenotype was reported [40]. As StaR is a repressor of *hfiA*, we predicted that overexpression of StaR should result in an increase in holdfast development. Indeed, *staR* overexpression results in a dramatic enhancement of visible holdfast (Figure 5F).

LovR is a single domain response regulator lacking a DNA-binding output domain [21], thus the effects of LovK-LovR on *hfiA* transcription must be indirect. We investigated whether inhibition of *hfiA* transcription by *lovK-lovR* is dependent on CtrA, GcrA, or StaR. Deletion of *staR* had no effect on inhibition of *hfiA* transcription by *lovK*-*lovR* (Figure 5C). We further demonstrated that *lovK-lovR* does not affect the occupancy of StaR at the *hfiA* locus (Figure S6). GcrA and CtrA regulated genes are not differentially regulated in *lovK-lovR* transcriptional profiling experiments [20] suggesting that the activity of GcrA or CtrA is not perturbed by LovK-LovR. Similarly, transcription from known GcrA and CtrA regulated promoters is not affected when *lovK* and *lovR* are coordinately overexpressed (Figure S6). We conclude that LovK and LovR affect *hfiA* transcription via a mechanism that is independent of StaR, GcrA, and CtrA.

### Holdfast development and *hfiA* transcription are regulated by the nutritional composition of the culture medium

**Figure 6:**
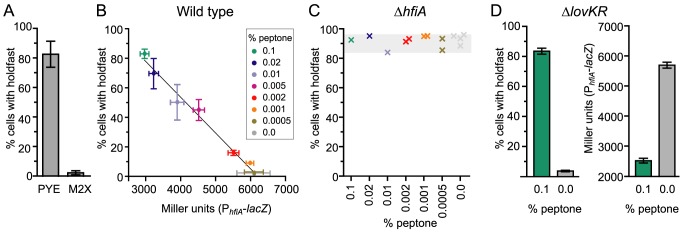
Nutrient environment affects *hfiA* transcription and the probability of holdfast development. **A.** Quantification of WGA-Alexa595 stained holdfast on wild-type CB15 cells grown in complex medium (PYE) or defined minimal medium (M2X). Bars represent the mean ± s.d. of 12 (PYE) or 18 (M2X) independent samples. At least 300 cells per sample were counted. **B.** Holdfast development and *hfiA* transcription are inversely correlated and dependent on the growth medium. Cultures grown in M2X supplemented with increasing amounts of peptone were assayed at a final density of 0.05–0.15 OD_660_. Holdfast were assessed as above in 4–8 independent cultures of wild-type cells per condition (mean ± s.e.m). *hfiA* transcription was assessed in 6–12 independent cultures of wild-type carrying the pRKlac290-P*_hfiA_* reporter plasmid per condition (mean ± s.e.m.). **C.** HfiA is required for nutrient dependent regulation of holdfast development. Holdfast were counted, as above, on Δ*hfiA* cultures grown M2X supplemented with a range of peptone concentrations. Each x represents holdfast counts in an individual culture of Δ*hfiA* cells colored by growth medium. **D.** Nutrient-dependent regulation of P*_hfiA_* and holdfast probability does not require *lovK* and *lovR*. As above, holdfast were assessed in Δ*lovKR* cells in 6 independent cultures per condition (mean ± s.e.m.). β-galactosidase activity was measured in Δ*lovKR* cells bearing the pRKlac290-P*_hfiA_* reporter plasmid (n = 10 independent cultures per condition; mean ± s.e.m).

The cell cycle expression profile of *hfiA* is coordinated with the timing of holdfast development. However, not every cell makes a holdfast. What determines whether a cell will elaborate a holdfast? Transcriptional profiling experiments suggest that culture environment affects *hfiA* transcription; in mid-log phase, cells grown in minimal medium (M2X) have 1.6 times more *hfiA* transcript than cells grown in complex medium (PYE) [43]. While this relative difference in *hfiA* transcript level is not large, *hfiA* is a highly expressed gene. Thus, the absolute difference in transcript levels is large. To test whether culture environment affects probability of holdfast development, we grew wild-type *C. crescentus* CB15 cells in either complex (PYE) or minimal defined medium (M2X) and quantified the fraction cells with visible holdfast. The culture environment has a dramatic impact on the probability that a cell displays a holdfast. In PYE medium, approximately 80% of cells develop a holdfast (Figure 6A) compared to 1–3% of cells in M2X.

We extended this analysis beyond these two standard growth media by analyzing both the probability of holdfast development and *hfiA* transcription from the P*_hfiA_*-*lacZ* reporter in a series of minimal media supplemented with increasing amounts of peptone, from 0.0005% to 0.1%. In this panel, we observed an approximately 2-fold change in activity from the *hfiA* promoter (Figure 6B). The probability of holdfast development in the population shows an inverse linear correlation with *hfiA* promoter activity (r^2^ = 0.99; Figure 6B). Cells cultured with little or no peptone exhibit the highest *hfiA* transcription and the lowest fraction of cells with holdfast. Increasing peptone concentration results in both decreased *hfiA* transcription and an increased fraction of cells with a holdfast. These data are consistent with a model in which modest relative changes in *hfiA* expression can have a large effect on holdfast development.

To test whether *hfiA* is required for regulated differences in holdfast between growth media, we evaluated holdfast development in the Δ*hfiA* null mutant. Regardless of the composition of the growth medium, Δ*hfiA* mutants elaborate a holdfast on nearly every cell (Figure 6C); only a small portion of swarmer cells can be found without holdfast. Transcription from the *hfsJ* promoter does not change between these growth conditions (Figure S7). We conclude that the capacity of a cell to elaborate a holdfast is controlled by the expression of the holdfast regulator, *hfiA*, and not by a change in expression of the holdfast synthesis gene *hfsJ*.

Finally, we tested if nutrient-dependent regulation of P*_hfiA_* and holdfast development requires the LovK-LovR sensory system. We measured the number of holdfast in strains lacking the *lovKR* locus. In minimal medium only small fraction of *ΔlovKR* cells display a holdfast. Upon supplementation with 0.1% peptone, the majority of *ΔlovKR* cells exhibit a holdfast (Figure 6D). Similarly, in a Δ*lovKR* background, the P*_hfiA_*-*lacZ* transcriptional reporter is reduced upon supplementation with 0.1% peptone (Figure 6D). Together these results indicate that LovK and LovR are not required for nutrient-dependent control of holdfast and suggest an additional, unknown regulator of *hfiA*.

## Discussion

**Figure 7:**
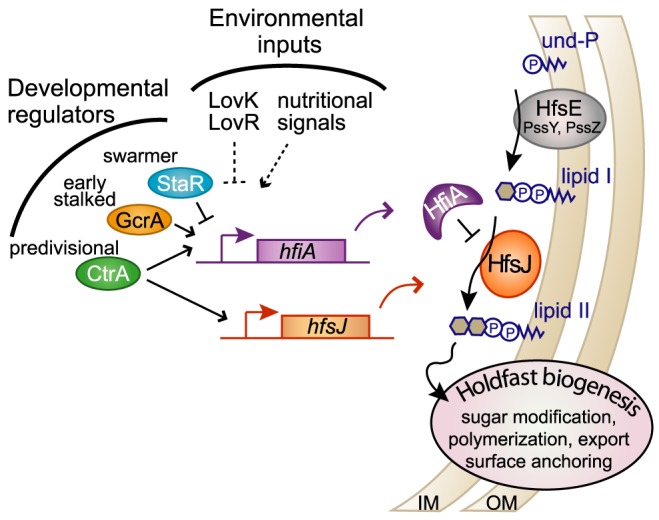
HfiA and HfsJ coordinately control holdfast development in response to cell cycle and environmental signals. Proposed model in which HfiA directly inhibits HfsJ, a WecG/TagA-family glycosyltransferase required for holdfast production. Expression of *hfsJ* and *hfiA* are controlled by cell cycle and environmental input signals. Solid and dashed lines indicate direct and indirect regulation respectively.

Holdfast adhesin development in *C. crescentus* is regulated by the developmental state of the cell and by the culture environment. This surface organelle emerges at the flagellated pole during the late swarmer cell stage ([8], [15]–[17], [19], Figure 1). We have discovered a novel small protein, HfiA, whose expression is developmentally and nutritionally regulated, and which functions as a potent inhibitor of holdfast. We demonstrate that the predicted glycosyltransferase, HfsJ, is a required component of the holdfast development machinery and that residues at the C-terminus of HfsJ mediate a direct interaction with HfiA. We propose a model in which HfiA functions as a cell cycle and nutritional checkpoint protein that prevents inappropriate holdfast development via post-translational inhibition of HfsJ (Figure 7).

Notably, the dynamic range of *hfiA* transcriptional control (∼2-fold) is modest at the population level compared to the dynamic range of holdfast probability (∼2-log). One prediction from this observation is that the binding affinity and cellular concentrations of HfiA and HfsJ are tuned such that this regulatory system is responsive to small changes rather than robust to large changes. This predication is consistent with a highly responsive and sensitive regulatory system.

HfsJ has strong similarity to WecG/TagA–family glycosyltransferases (E-value<e^−84^) [27]. Enzymes in this family are known to catalyze the transfer of a nucleotide diphosphate (NDP)-activated sugar to monoglycosylated Und-PP (i.e. lipid I) [44], [45]. The product of this reaction is the phosphoglycolipid, Und-PP-disaccharide (i.e. lipid II). Varied forms of lipid II are precursors for extracellular polysaccharide structures in bacteria including lipopolysaccharide, wall teichoic acid, capsular polysaccharide, and holdfast. In *C. crescentus*, there is apparent redundancy in the holdfast synthesis enzymes predicted to catalyze formation of lipid I [12]. Our genetic data suggest that HfsJ is solely responsible for the production of holdfast lipid II. *B. subtilis* and *Staphylococcus aureus* TagA catalyze a specific transformation of lipid I to lipid II that commits the phosphoglycolipid for wall teichoic acid biosynthesis [46]. By analogy, we predict that HfsJ commits its phosphoglycolipid substrate to holdfast biosynthesis. Post-translational regulation of such a “gate-keeping” enzyme would enable specific control of lipid I commitment to holdfast development (Figure 7).

Temporally staggered cell-cycle transcription of *hfiA* and *hfsJ* correlates with the developmental timing of holdfast synthesis. Key developmental regulators physically interact with and regulate both of these genes, directly tying holdfast development to the core cell cycle control network. The master cell cycle regulator, CtrA, activates transcription of both *hfiA* and *hfsJ*. The methylation-responsive transcriptional regulator, GcrA [41], and the developmental regulator, StaR, provide additional layers of direct *hfiA* regulation. The activities of these regulators on *hfiA*, but not *hfsJ* can account for the temporal shift in *hfsJ* and *hfiA* expression. Consistent with the observation of *hfs* gene transcripts in late pre-divisional and swarmer cells ([31]–[33], Figure 5), swarmer cells are born preloaded with all the proteins required to synthesize a holdfast [17], [19]. *staR* is activated in swarmer cells ([31]–[33], [47], Figure 5) and as a functional repressor of *hfiA*, presumably drives the decrease in *hfiA* transcription prior to holdfast development. Decreased expression of the holdfast inhibitor is predicted to permit holdfast production as cells approach the swarmer-to-stalk cell transition. Upon this transition, cells accumulate GcrA [38], which can initiate activation of *hfiA* expression, but not *hfsJ*. As the cell cycle progresses, de novo synthesis and activation of CtrA [37] should reinforce expression of the inhibitor and also activate *hfsJ* in preparation for the next generation swarmer cell.

Notably, *hfsJ* is among the last holdfast synthesis genes to be transcriptionally activated; it does not reach maximum transcription until just prior or coincident with cell division (Figure 5). This delayed expression provides two intuitive mechanisms that should restrict premature holdfast synthesis. First, HfsJ is essential for holdfast biosynthesis, thus a preassembled machine will not be functional in the predivisional cell until *hfsJ* is expressed, just around the time of cell division. Second, peak *hfiA* expression precedes that of its target. A pool of accumulated inhibitor should block activity of nascent HfsJ in the late predivisional cell. Together these features ensure that the motile swarmer cells are not born with a holdfast and are able to fulfill a dispersal role.

What, then, relieves HfiA inhibition so that holdfast development can progress in the swarmer cell? One possibility is that HfiA is inherently unstable and rapidly degraded by cellular proteases. Indeed, proteins optimized for regulatory flexibility tend to have short half-lives [48]. If HfiA is unstable, high synthesis rates would be necessary to maintain an appreciable steady state concentration in the cell. In this scenario, the initial concentration of HfiA in the swarmer cell (where *hfiA* is not transcribed) could serve as a timer for the initiation of holdfast synthesis. Several efforts by our research group to quantify HfiA protein levels in the cell have been unsuccessful. These negative results provide indirect support for the hypothesis that HfiA is an unstable polypeptide, though we still seek direct experimental support for this hypothesis.

Alternatively, post-translational modification could affect HfiA stability or its binding affinity with HfsJ. Surface contact-dependent perturbation of the flagellum [19] could serve as a signal for HfiA modification or degradation. Another possibility is that cyclic-di-GMP (cdG) could serve as a second messenger that directly or indirectly inactivates HfiA or activates HfsJ. In many systems cdG serves as a developmental cue signaling the transition from motile to non-motile states [49]. Indeed the activity of the diguanylate cyclase, PleD, is cell cycle regulated and activated during the swarmer to stalk transition [50]; *C. crescentus* cells lacking *pleD* are delayed in holdfast development [17]. Thus, it is reasonable to predict that cdG may play a role in control of the HfiA-HfsJ adhesion checkpoint.

While the developmental circuitry of the cell directly controls *hfiA* expression, environmental signals provide an additional regulatory input that can override developmental control. A mixed population of cells grown in carbon replete minimal defined medium have 60% more *hfiA* transcript than cells grown in complex medium [43]; this correlates with the observed frequency of holdfast-bearing cells in a population (i.e. cells grown in minimal medium rarely elaborate a holdfast while the majority cells grown in complex medium possess holdfast) (Figure 6). Moreover, supplementation of minimal defined medium with peptone modulates both *hfiA* expression over a two-fold range and the probability of holdfast development over a 2-log range (Figure 6). A similar correlation is observed upon overexpression of the LovK-LovR two-component sensory system, which results in *hfiA* repression and increased probability of holdfast development (Figures 1 & 2). Notably, the nutrient-dependent control of *hfiA* transcription and holdfast development is independent of the LovK-LovR sensory system. The exact regulatory connection between *hfiA* transcription and either LovK-LovR signaling or the metabolic state of the cell remains unclear. Indeed, the data presented here speak to existence of at least one additional direct regulator of *hfiA*, as the repressive effect of LovK-LovR on *hfiA* transcription is necessarily indirect and also independent of CtrA, GcrA, and StaR.

Given the permanence of the cellular decision to adhere to a surface, it is not surprising that environmental and nutritional stimuli influence *hfiA* expression and holdfast adhesin development. Our study provides evidence that multiple developmental and environmental signals are integrated at the promoter of *hfiA*, which encodes a novel, small protein inhibitor of the required holdfast synthesis enzyme, HfsJ. *C. crescentus* employs a multi-level regulatory system that ensures proper timing of holdfast development, and safeguards against permanent cell adherence in a sub-optimal environment.

## Materials and Methods

### Strain construction and growth conditions

Standard cloning methods, strain construction techniques and growth conditions were employed and are detailed in the Text S1. Strains and primers used are in Table S6.

### Microscopy

Cells were imaged with a DM5000 microscope (Leica) in phase contrast and fluorescence modes using a HCX PL APO 63×/1.4na Ph3 objective. Fluorescent samples were excited with an external mercury halide bulb in an EL6000 lamp (Leica). Standard filter sets were used to detect WGA-Alexa594 (Chroma set 41043) and the fluorescent protein, Venus (Chroma set 41028). Images were captured using an Orca-ER digital camera (Hamamatsu) controlled by Image-Pro (Media Cybernetics, Inc.).

### Holdfast stain

To visualize holdfast, 100–500 µl of cells were incubated for 10–15 minutes with 50 µg/ml Wheat Germ Agglutinin, Alexa Fluor 594 Conjugate (Life Technologies, Molecular Probes), diluted with 1 ml water or media, collected by centrifugation for 3 minutes (14,000 g), and resuspended in 20–50 µl. For quantitative analyses, overnight cultures were diluted to an approximate OD_660_ of 0.00005 so that after 16–18 hours of growth the culture density was between 0.05 and 0.1 OD_660_. This approach minimized cell-cell adhesion and rosette formation and ensured that all cells were “born” into nutritionally replete conditions.

### Transcriptional analysis

Global transcriptional profiling of Δ*sigT xylX*::pMT585 *vanR*::pMT528 (EV) and Δ*sigT xylX*::pMT585-*lovR vanR*::pMT528-*lovK* cultures were conducted as in [20]. β-galactosidase activity from promoter-*lacZ* fusions was measured colorimetrically [51] as in [20].

### 5′ RACE

Transcription start sites were identified by mapping 5′ ends of mRNA using the FirstChoice RLM-RACE kit (Life Technologies, Ambion) following the manufacturer's protocol. The RNA template was extracted from log phase cells grown in M2X medium using Trizol (Life Technologies, Invitrogen). 5′GTCGGTCGTGCGCATAGT and 5′GATCTTCGAGCGGCGAAA primers were used as *hfiA* specific primers.

### ChIP-seq

Mid-log phase cells grown in PYE were cross-linked in 10 mM sodium phosphate (pH 7.6) and 1% formaldehyde at room temperature for 10 min and on ice for 30 min thereafter, washed three times in phosphate buffered saline (PBS) and lysed in a Ready-Lyse lysozyme solution (Epicentre, Madison, WI) according to the manufacturer's instructions. Lysates were sonicated (Sonifier Cell Disruptor *B*-*30*; Branson) on ice using 10 bursts of 20 sec at output level 4.5 to shear DNA fragments to an average length of 300–500 bp and cleared by centrifugation for 2 minutes (14,000 rpm, 4°C). Lysates were normalized by protein content, diluted to 1 mL using ChIP buffer (0.01% SDS, 1.1% Triton X-100, 1.2 mM EDTA, 16.7 mM Tris-HCl (pH 8.1), 167 mM NaCl plus protease inhibitors (Roche, Switzerland) and pre-cleared with 80 µL of protein-A agarose (Roche, Switzerland) and 100 µg BSA. Ten percent of the supernatant was removed and used as total chromatin input DNA.

Polyclonal antibodies to StaR or CtrA were added to the remains of the supernatant (1∶1,000 dilution), incubated overnight at 4°C with 80 µL of protein-A agarose beads pre-saturated with BSA, washed once with low salt buffer (0.1% SDS, 1% Triton X-100, 2 mM EDTA, 20 mM Tris-HCl (pH 8.1), 150 mM NaCl), high salt buffer (same as previous with 500 mM NaCl) and LiCl buffer (0.25 M LiCl, 1% NP-40, 1% sodium deoxycholate, 1 mM EDTA, 10 mM Tris-HCl (pH 8.1)) and twice with TE buffer (10 mM Tris-HCl (pH 8.1) and 1 mM EDTA). The protein•DNA complexes were eluted in 500 µL freshly prepared elution buffer (1% SDS, 0.1 M NaHCO_3_), supplemented with NaCl to a final concentration of 300 mM and incubated overnight at 65°C to reverse the crosslinks. The samples were treated with 2 µg of Proteinase K for 2 h at 45°C in 40 mM EDTA and 40 mM Tris-HCl (pH 6.5). DNA was extracted using phenol∶chloroform∶isoamyl alcohol (25∶24∶1), ethanol-precipitated using 20 µg of glycogen as carrier and resuspended in 100 µL of water.

Illumina Genome Analyzer IIx or HiSeq 2000 runs of barcoded ChIP-Seq libraries yielded several million reads that were mapped to *C. crescentus* NA1000 (NC_011916) using the ELAND alignment algorithm (services provided by Fasteris SA, Switzerland). Analysis of sequences is described in Text S1.

### HfiA suppressor screen

The goal of this screen was to identify mutants that are insensitive to HfiA and can develop holdfast even when *hfiA* is overexpressed. Our strategy to enrich the population with holdfast+ mutants is conceptually the opposite of that used by [7] to enrich a population with holdfast null mutants by removing holdfast bearing cells with cheese cloth. Here unattached cells are removed by aspiration and surface attached cells are allowed to populate the culture.

FC1935 and FC1936, strains that overexpress *hfiA* from either the xylose-inducible promoter on a mid-copy replicating plasmid or from the xylose promoter integrated at two independent sites on the chromosome, respectively, were used as the parental strains. Enrichment was carried out in complex medium, which promotes holdfast development in wild-type cells, supplemented with xylose to induce overexpression of *hfiA*. Explicit mutagenesis was unnecessary; spontaneous mutants arise in the course of the enrichment.

Starter cultures inoculated from freshly grown colonies into PYE supplemented with 0.15% xylose and appropriate antibiotics were diluted 1∶100 in 1 ml of fresh medium in each well of a sterile 24-well polystyrene plate with a lid. The lid was sealed with a strip of AeraSeal air-permeable sealing film (Excel Scientific) to prevent evaporation. Plates were incubated with gentle shaking (155 rpm) at 30°C overnight. The culture medium was removed by aspiration. Unattached cells were thoroughly washed away with a stream of sterile water expelled from a 20 cc syringe through a 22 G needle. The inoculum of attached cells was allowed to regrow to saturation in 1 ml of fresh medium under the same growth conditions. Washing and regrowth in fresh medium were repeated. After the first wash, the wells appear clear and regrowth requires 36–48 hours, but after 3–4 rounds of washing the wells are cloudy with attached cells and the outgrowth saturates in less than 18 hours. The culture was serially diluted and plated on solid medium to isolate single colonies.

Isolated colonies were subjected to several secondary screens. First, the P_xyl_-*hfiA* region of the overexpression plasmids were amplified and re-sequenced to eliminate mutants in the overexpression system. Second, surface attachment to polystyrene was measured with crystal violet staining (see below) to confirm enhanced adhesion capacity of each isolate. Third, cells were grown in minimal medium with xylose as the sole carbon source (M2X) to ensure a functional xylose transport system. Fourth, WGA-Alexa594 binding was used to assess holdfast development.

### Whole genome sequencing

Genomic DNA was isolated from individual suppressor strains using guanidium thiocyanate [52]. Bar-coded Next Generation Sequencing libraries were pooled and sequenced (50-bp single end reads) using the SOLiD 5500 xl sequencing platform (Applied Biosystems, Life Technologies) generating an average of 12 million reads per library. The Functional Genomics Facility at the University of Chicago provided sequencing services. Sequences were processed through an automated analysis pipeline by University of Chicago Center for Research Informatics. The analysis pipeline is described in more detail in Text S1.

### Surface attachment

Cells were grown from 5 ul of an overnight starter culture in 1 ml of fresh growth medium in 24-well sterile polystryrene plates with lids. Lids were sealed with AeraSeal air-permeable sealing film (Excel Scientific) and plates were incubated with gentle shaking (155 rpm) at 30°C for 24 hours. Culture medium and planktonic cells were removed by aspiration and washed away with tap water. Surface attached cells were measured by crystal violet staining using a protocol similar to those outlined by [53], [54]. Briefly, wells were incubated with 1 ml 0.01% crystal violet for 5 minutes with gentle shaking then washed with tap water to remove unbound stain. Bound stain was extracted with 1.5 ml 95% ethanol while gently shaking for 5–10 minutes. Extracted stain was diluted 1∶6 and the optical density at 575 nm was measured spectroscopically.

### Protein co-expression and co-purification

A 50 ml overnight culture (30°C, 220 rpm) was used to inoculate 500 ml of LB broth supplemented with appropriate antibiotics and 0.2% glucose to repress expression of endogenous maltose degradation genes. When the culture reached OD_660_∼0.8, 0.5 mM of IPTG was added to induce expression. After 2 hours (30°C, 220 rpm), cells were harvested by centrifugation for 20 min (12,000 g, 4°C) and the pellet was resuspended in 5 ml of Tris-NaCl buffer (10 mM Tris pH 7.4, 150 mM NaCl) supplemented with 10 mM imidazole, 5 µg/ml of DNase I and PMSF. Cells were disrupted by three passages in a French pressure cell, and cell debris was removed by centrifugation for 20 min (25,000 g, 4°C). The supernatant was then mixed with 500 µl of Amylose resin (New England Biolabs) pre-equilibrated with Tris-NaCl buffer, which allowed for binding of MBP domains. The resin was thoroughly washed with the Tris-NaCl buffer and bound proteins were eluted with 500 µl of Tris-NaCl buffer supplemented with 20 mM maltose. The eluted proteins were mixed with 100 µl of Ni^2+^-NTA Sepharose affinity resin (GE Life Sciences) pre-equilibrated with Tris-NaCl buffer to allow binding of His-tagged proteins. Two stringent washing steps were performed with Tris-NaCl buffer containing 10 mM or 75 mM imidazole followed by elution with 100 µl of 1 M imidazole Tris-NaCL buffer. To monitor proteins bound and eluted from each resin, samples were separated by electrophoresis on a 14% SDS-PAGE gel and stained with Coomassie. Polyacrylamide fragments containing purified proteins were excised and sent to the Pan Facility at Stanford University, Palo Alto, CA for Mass Mapping to confirm protein identity. In a similar way, the reverse experiment (purification first with Ni^2+^ Sepharose affinity resin and then with Amylose resin) was also performed.

### Bacterial two-hybrid assay

Based on the system developed by [30], plasmid bearing fusions to either the T18 or T25 domains of adenylate cyclase were co-transformed into the adenylate cyclase null strain, BTH101, by electroporation. The outgrowth was serially diluted and plated on LB-agar containing Amp_100_, Kan_50_, X-gal (40 µg/ml) and IPTG (0.5 mM). The color of single colonies from each transformation was evaluated after 48 hours growth at 30°C. Two colonies from each strain were repatched on identical medium for side-by-side comparisons.

## Supporting Information

Figure S1 *Caulobacter* cell aggregation is modulated by *lovK*-*lovR* in a holdfast (*hfsA*) dependent manner. **A.** When cultured on an angle in a roller, aggregated cells accumulate in a ring at the lip of culture. To quantify bulk accumulation of cell aggregates, cultures were inoculated from fresh plates into M2 medium supplemented with xylose and vanillate. After overnight growth, cells were diluted in 5 ml of fresh medium to an OD_660_ of 0.05 and grown in a roller for exactly 24 hours. The culture medium was removed by aspiration and cells remaining in the tube, loosely associated with the glass were resuspended in 1.5 ml fresh medium by vortexing. The OD_660_ of the resuspended cells was then measured. **B.** Images of the culture tubes containing the strains shown in Figure 1B in the main text bearing either empty plasmids (EV) or *lovK* and *lovR* inducible overexpression plasmids. Arrows indicate position of rings. **C.** Quantification of ring accumulated cells for the genotypes in (B). Data represent 9 independent cultures assayed on three different days. Values are normalized to the mean wild-type EV cultures on each day. Bars indicate mean ± s.d. Means were compared with one-way ANOVA followed by Tukey's post-test. Rings from wild-type *lovK* ^++^ *lovR* ^++^ and Δ*sigT lovK* ^++^ *lovR* ^++^ cultures are different from wild-type empty vector control (p<0.001) and not significantly different from each other (p>0.05).
(JPG)

Figure S2 Molecular characterization of the *hfiA* locus. **A.** 5′ end of the *hfiA* locus and translational fusions with *lacZ*. Green type indicates NTG codons that could function as translation start sites (annotated a-e). Triplets indicate predicted coding sequence. Uppercase letters indicate reannotated coding sequence starting at putative start ‘c’. **B.** β-galactosidase activity from *lacZ* translational fusions including putative starts a and b or putative starts a, b, c, d and e (shown in (A)). Data represent mean ± s.d. of 8 independent samples assayed over 3 different days. **C.** Quantitative analysis of holdfast in cells bearing chromosomal mutations in one or multiple putative translation start codons. Bases mutated in each codon are shown in blue. Cells were grown in M2X medium and holdfast were visualized with WGA-Alexa594. Bars represent mean ± s.d. of 3 independent samples. At least 300 cells were counted in each sample.
(JPG)

Figure S3 Positions of plasmid encoded intragenic *hfiA* suppressing mutations. Wild-type *hfiA* sequence cloned into the xylose-inducible overexpression plasmid, pMT805. Genome coordinates for the reannotated translation start site are indicated. Blue highlight: site of nonsense SNPs. Yellow highlight: site of non-synonymous SNP. Green highlight: site of insertion. Dots above: duplicated sequence. Underlined: deleted sequence.
(JPG)

Figure S4 Molecular characterization of *hfsJ*, a putative glycosyltransferase gene required for holdfast development. **A.** 5′ end of *hfsJ*. The CB15 and NA1000 genome annotations predict different translational start codons for *hfsJ* (in green, indicated by *a* and *b* respectively); the resulting protein predicted by the CB15 annotation (CC_0095) is 10 residues longer than predicted in the NA1000 annotation (CCNA_00094). Mutation of the translation start site should result in a strain that phenocopies an in-frame deletion strain (Δ*hfsJ*). To test each putative start codon, we built allele replacement strains in which each putative start codon was mutated from ATG to ATA (below the translation). Data support a model in which translation initiates at codon ‘b’ (indicated by the orange shading and the uppercase type). **B–D.** Bulk surface adhesion measured by crystal violet staining of attached cells after growth in 24-well polystyrene plates. Each bar represents mean ± s.d. of at least 4 independent assays. **E–G.** WGA-Alexa594 lectin staining of holdfast. Cells were grown in PYE and diluted so that after 15 hours of outgrowth, cultures would be in early log phase (between 0.05–0.15 OD_660_) for staining. (**B,E**) Mutation of putative start codon ‘a’ does not affect the surface adhesion or holdfast phenotype. Mutation of codon ‘b’ ablates surface adhesion and holdfast synthesis, similar to the *ΔhfsJ* in-frame deletion strain. (**C,F**) The surface adhesion and holdfast defects of the Δ*hfsJ* null strain can be complemented by a plasmid encoded copy of *hfsJ* expressed from an inducible promoter. EV = empty vector control. (**D,G**) The surface adhesion and holdfast defects of the Δ*hfsJ* null strain cannot be complemented by plasmid encoded copies of the related *B. subtilis tagA* or *E. coli wecG* glycosyltransferases. EV = empty vector control. Notably, expression of *E. coli* WecG in *C. crescentus* alters cell morphology resulting in a decrease in cell curvature. These cells still exhibit stalks and motility. **H.** HfsJ-venus is distributed throughout the cell. The Δ*hfsJ* in-frame deletion strain was transformed with a suicide plasmid encoding an HfsJ-venus fluorescent protein fusion expressed from the native *hfsJ* promoter. The resulting strain, CB15 Δ*hfsJ*::pMT666-P*_hfsJ_*-*hfsJ*-venus, was grown in PYE overnight, diluted in fresh medium and outgrown for 1 hour before imaging the cells. The fluorescence signal is not localized to the pole, but is distributed throughout the cell. **I.** Holdfast staining as in B–D demonstrates that the *hfsJ*-venus fusion in H functionally complements the Δ*hfsJ* holdfast-null phenotype. **J.** Western blot using anti-GFP monoclonal antibodies to detect the venus variant of GFP. Cells were lysed by French press and fractionated by centrifugation. The supernatant and pellet fractions from wild-type and ΔhfsJ::pMT666-P*_hfsJ_*-*hfsJ*-venus cell lysates were separated on a 4–20% gradient SDS-PAGE gel. Molecular weight standards are indicated. HfsJ-venus is indicated with a pink asterisk.
(JPG)

Figure S5 HfiA – HfsJ co-expression and co-purification controls. Plasmids used and proteins expressed are indicated above each SDS-PAGE gel. Aliquots of purified proteins are loaded left to right reflecting sequential purification steps. Relevant bands are indicated by black arrowheads. **A.** Co-expression and purification similar to that described in the main text, but in the reverse order, starting with Ni^2+^ resin to capture His_6_-HfsJ followed by amylose resin to capture MBP-HfiA. Increased non-specific binding to Ni^2+^ resin in step 1 results in reduced final purity of isolated proteins. Nevertheless, co-purification is observed after both Ni^2+^ and amylose resin purification steps. **B.** When MBP-HfiA is expressed from a pMal-c2x plasmid, MBP-HfiA along with a protein the size of MBP lacking the HfiA fusion elute from amylose resin as expected. However, neither of these species bind and elute from Ni^2+^ resin indicating that co-purification is not mediated by an interaction between MBP or HfiA with the Ni^2+^ resin. **C.** MBP without the HfiA fusion does not co-purify with co-expressed His_6_-HfsJ. His_6_-HfsJ is not detected after affinity purification using amylose resin or enriched in a second round of purification with Ni^2+^ resin. **D.** MBP without the HfiA fusion does not co-purify with His_6_-HfsJ. Together the results in B, C and D indicate that co-purification of His_6_-HfsJ and MBP-HfiA is mediated by an interaction between HfsJ and HfiA, and not by spurious interactions with MBP or the purification resins.
(JPG)

Figure S6 StaR, CtrA and GcrA promoter binding and transcriptional regulatory activities are not affected by *lovK*-*lovR* overexpression. **A–B.** Overexpression of *lovK* and *lovR* does not influence the efficiency of StaR precipitation of P*_hfiA_*. StaR-ChIP followed by qPCR on DNA precipitated from WT, Δ*staR*, *lovK-lovR* overexpression or vector control (EV) strains. Primers amplified the *hfiA* promoter region (**A**) or the *pilA* promoter region (**B**) as a negative control region that is not occupied by StaR. Real-time PCR was performed using a Step-One Real-Time PCR system (Applied Biosystems, Foster City, CA) using 5 µL of each ChIP sample in a reaction with SYBR green PCR master mix (Quanta Biosciences, Gaithersburg, MD). Standard curve generated from the cycle threshold (Ct) value of the serially diluted chromatin input was used to calculate the percentage input value of each sample. Average values are from triplicate measurements done per culture. The final data were generated from three independent cultures. The DNA regions analyzed by real-time PCR were from nucleotide −147 to +126 relative to the start codon of *hfiA* and from −287 to −91 relative to the start codon of *pilA* with the following primers: *hfiA* ChIP F2- 5′AAACCACAACAACGAGGCCAA; *hfiA* ChIP R2- 5′ACGGACGTGATGCACTACAGCTA; *pilA* ChIP F- 5′CGACTGCACTTAATGGCCAG; and *pilA* ChIP R- 5′GCCAGCATCACTTTCTTTGG. **C.** β-galactosidase activity from transcriptional fusions between known CtrA or GcrA regulated promoters and *lacZ* was evaluated in strains overexpressing *lovK* and *lovR* (dark grey) and in empty vector (EV) control strains (light grey). Promoters assayed are indicated on the x-axis. No significant differences were observed upon *lovK-lovR* overexpression.
(JPG)

Figure S7 *hfsJ* transcription is not significantly affected by the nutrient content of the culture medium. β-galactosidase activity from the P*_hfsJ_*-*lacZ* transcriptional fusion (pRKlac290-P*_hfsJ_*) was measured in wild-type cells grown in M2X defined minimal medium supplemented with increasing amounts of peptone. As in Figure 6, starter cultures were diluted to a low OD so that after ∼16 hours of growth, the OD_660_ of the culture was ∼0.1. Dots represent individual measurements from independent cultures collected over 2 different days colored as in Figure 6. Black lines represent the mean ± s.e.m. Differences between conditions were statistically assessed with ANOVA followed by Tukey's multiple comparison post-test. No significant (p<0.05) differences were found.
(JPG)

Table S1 Spontaneous mutations that suppress the *hfiA* overexpression phenotype.
(PDF)

Table S2 StaR ChIP-seq top hits.
(XLSX)

Table S3 StaR ChIP-seq read depth compiled for 50 bp windows of the NA1000 genome (GHA16_StaR).
(XLSX)

Table S4 CtrA ChIP-seq top hits.
(XLSX)

Table S5 CtrA ChIP-seq read depth compiled for 50 bp windows of the NA1000 genome (GHA17_CtrA).
(XLSX)

Table S6 Plasmids, primers and strains used.
(PDF)

Text S1 Supplemental experimental procedures.
(PDF)
