# Supplementary figures and images for: A Cell Cycle and Nutritional Checkpoint Controlling Bacterial Surface Adhesion
Source: PLoS Genet. 2014 Jan 23;10(1):e1004101. doi: 10.1371/journal.pgen.1004101 (PMC3900383; doi:10.1371/journal.pgen.1004101)

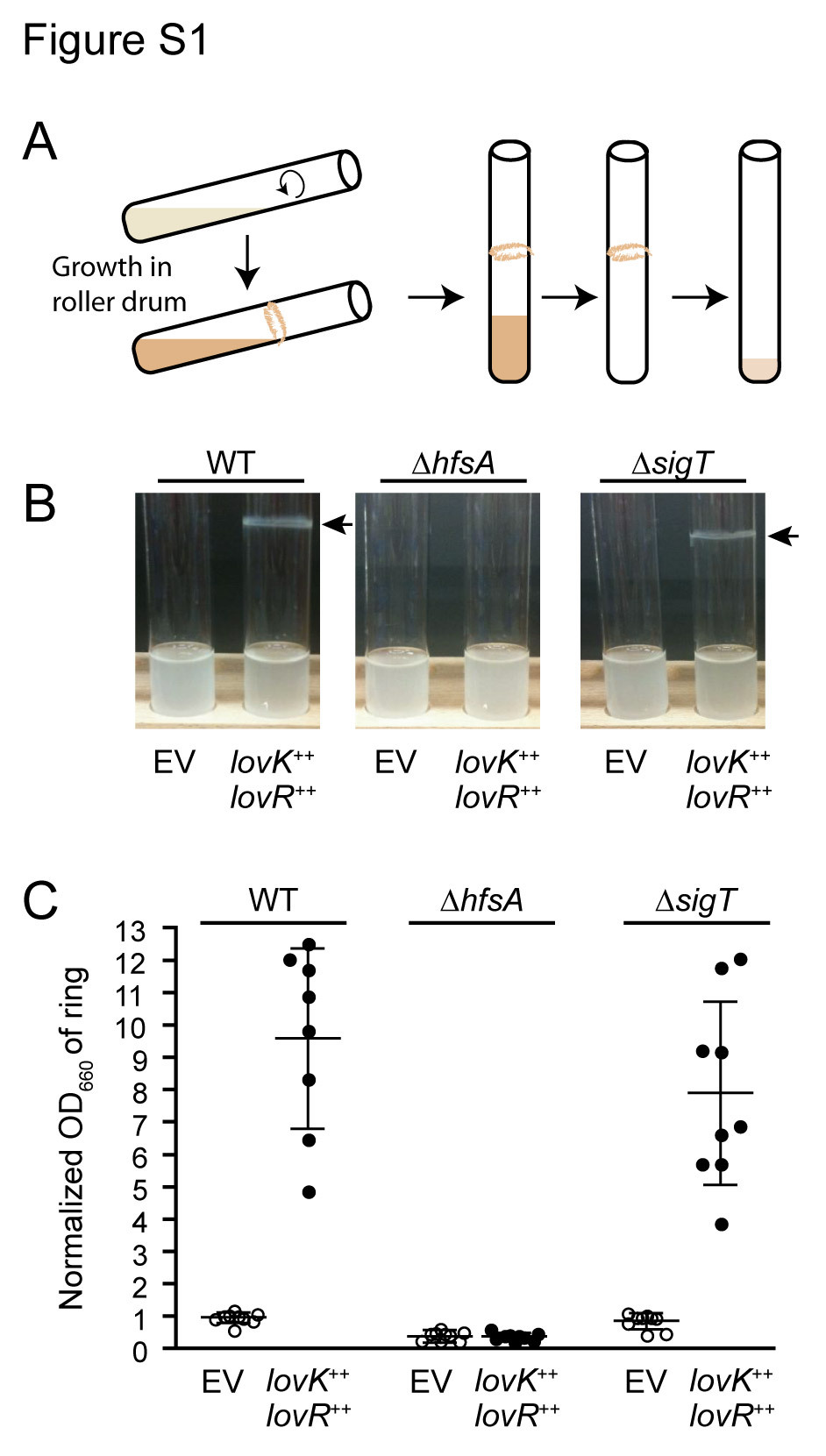

Supplement: Figure S1 — Caulobacter cell aggregation is modulated by lovK-lovR in a holdfast (hfsA) dependent manner. A. When cultured on an angle in a roller, aggregated cells accumulate in a ring at the lip of culture. To quantify bulk accumulation of cell aggregates, cultures were inoculated from fresh plates into M2 medium supplemented with xylose and vanillate. After overnight growth, cells were diluted in 5 ml of fresh medium to an OD660 of 0.05 and grown in a roller for exactly 24 hours. The culture medium was removed by aspiration and cells remaining in the tube, loosely associated with the glass were resuspended in 1.5 ml fresh medium by vortexing. The OD660 of the resuspended cells was then measured. B. Images of the culture tubes containing the strains shown in Figure 1B in the main text bearing either empty plasmids (EV) or lovK and lovR inducible overexpression plasmids. Arrows indicate position of rings. C. Quantification of ring accumulated cells for the genotypes in (B). Data represent 9 independent cultures assayed on three different days. Values are normalized to the mean wild-type EV cultures on each day. Bars indicate mean ± s.d. Means were compared with one-way ANOVA followed by Tukey's post-test. Rings from wild-type lovK ++ lovR ++ and ΔsigT lovK ++ lovR ++ cultures are different from wild-type empty vector control (p<0.001) and not significantly different from each other (p>0.05). (JPG) [file pgen.1004101.s001.jpg]

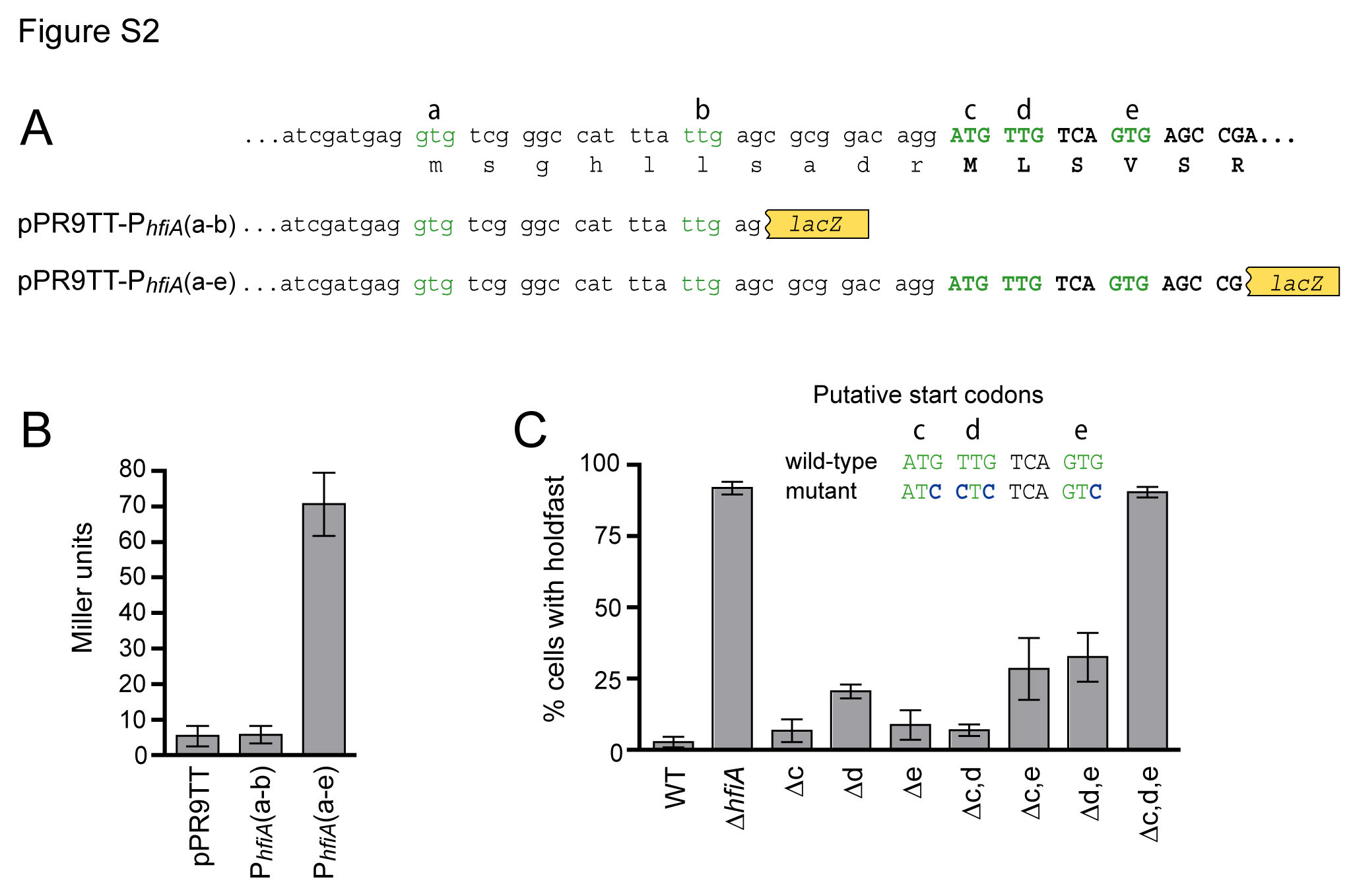

Supplement: Figure S2 — Molecular characterization of the hfiA locus. A. 5′ end of the hfiA locus and translational fusions with lacZ. Green type indicates NTG codons that could function as translation start sites (annotated a-e). Triplets indicate predicted coding sequence. Uppercase letters indicate reannotated coding sequence starting at putative start ‘c’. B. β-galactosidase activity from lacZ translational fusions including putative starts a and b or putative starts a, b, c, d and e (shown in (A)). Data represent mean ± s.d. of 8 independent samples assayed over 3 different days. C. Quantitative analysis of holdfast in cells bearing chromosomal mutations in one or multiple putative translation start codons. Bases mutated in each codon are shown in blue. Cells were grown in M2X medium and holdfast were visualized with WGA-Alexa594. Bars represent mean ± s.d. of 3 independent samples. At least 300 cells were counted in each sample. (JPG) [file pgen.1004101.s002.jpg]

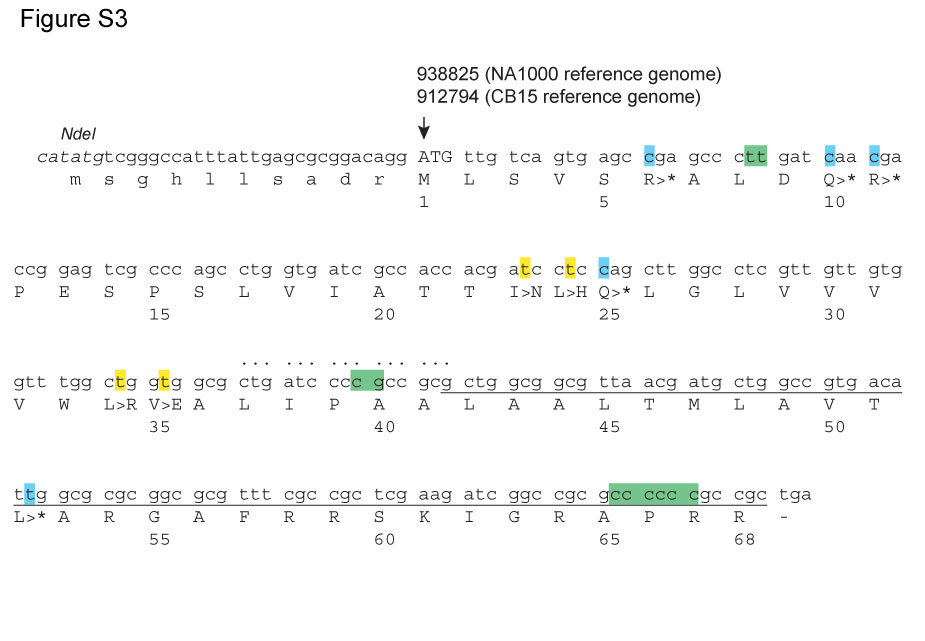

Supplement: Figure S3 — Positions of plasmid encoded intragenic hfiA suppressing mutations. Wild-type hfiA sequence cloned into the xylose-inducible overexpression plasmid, pMT805. Genome coordinates for the reannotated translation start site are indicated. Blue highlight: site of nonsense SNPs. Yellow highlight: site of non-synonymous SNP. Green highlight: site of insertion. Dots above: duplicated sequence. Underlined: deleted sequence. (JPG) [file pgen.1004101.s003.jpg]

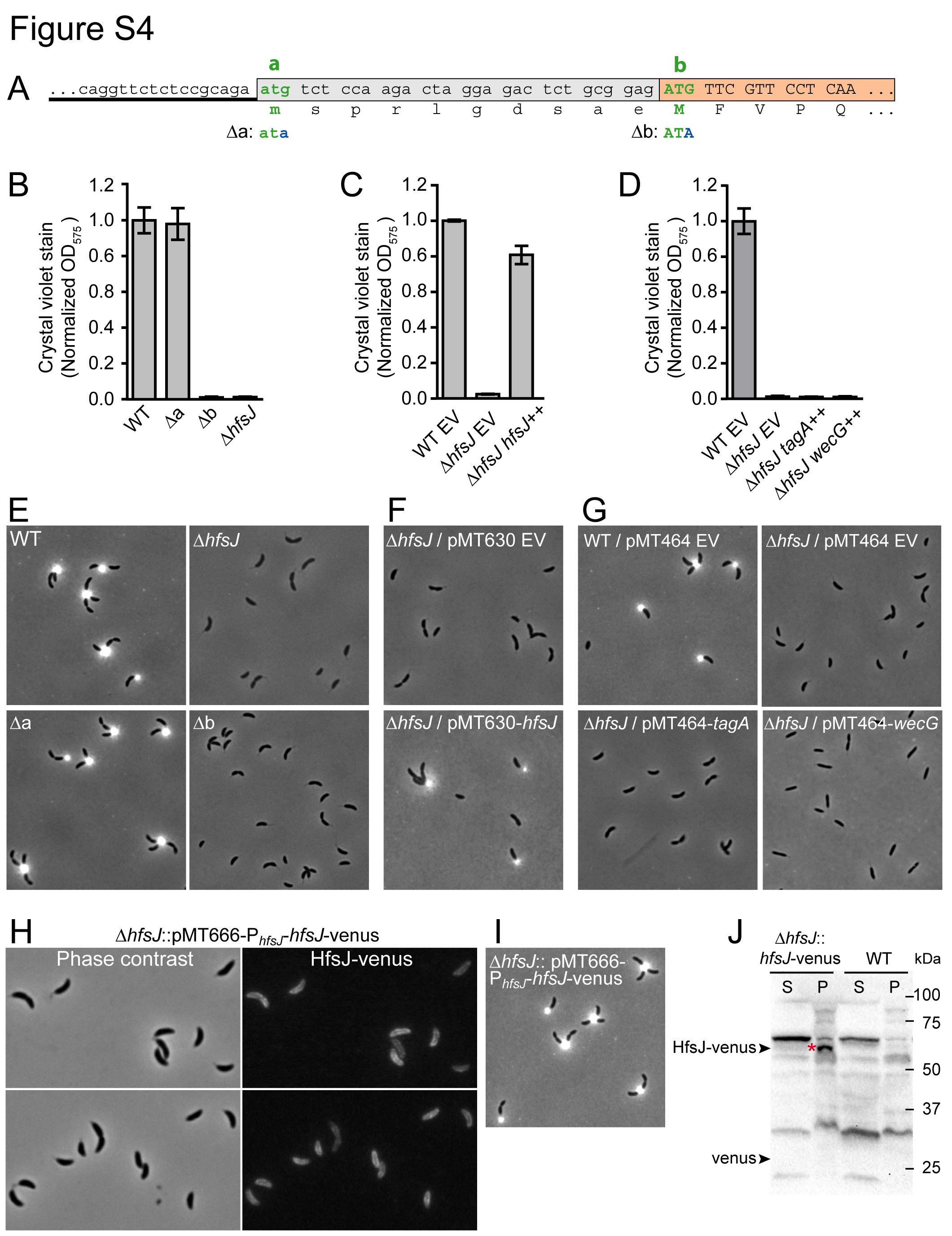

Supplement: Figure S4 — Molecular characterization of hfsJ, a putative glycosyltransferase gene required for holdfast development. A. 5′ end of hfsJ. The CB15 and NA1000 genome annotations predict different translational start codons for hfsJ (in green, indicated by a and b respectively); the resulting protein predicted by the CB15 annotation (CC_0095) is 10 residues longer than predicted in the NA1000 annotation (CCNA_00094). Mutation of the translation start site should result in a strain that phenocopies an in-frame deletion strain (ΔhfsJ). To test each putative start codon, we built allele replacement strains in which each putative start codon was mutated from ATG to ATA (below the translation). Data support a model in which translation initiates at codon ‘b’ (indicated by the orange shading and the uppercase type). B–D. Bulk surface adhesion measured by crystal violet staining of attached cells after growth in 24-well polystyrene plates. Each bar represents mean ± s.d. of at least 4 independent assays. E–G. WGA-Alexa594 lectin staining of holdfast. Cells were grown in PYE and diluted so that after 15 hours of outgrowth, cultures would be in early log phase (between 0.05–0.15 OD660) for staining. (B,E) Mutation of putative start codon ‘a’ does not affect the surface adhesion or holdfast phenotype. Mutation of codon ‘b’ ablates surface adhesion and holdfast synthesis, similar to the ΔhfsJ in-frame deletion strain. (C,F) The surface adhesion and holdfast defects of the ΔhfsJ null strain can be complemented by a plasmid encoded copy of hfsJ expressed from an inducible promoter. EV = empty vector control. (D,G) The surface adhesion and holdfast defects of the ΔhfsJ null strain cannot be complemented by plasmid encoded copies of the related B. subtilis tagA or E. coli wecG glycosyltransferases. EV = empty vector control. Notably, expression of E. coli WecG in C. crescentus alters cell morphology resulting in a decrease in cell curvature. These cells still exhibit stalks and motility. H. Hfs [file pgen.1004101.s004.jpg]

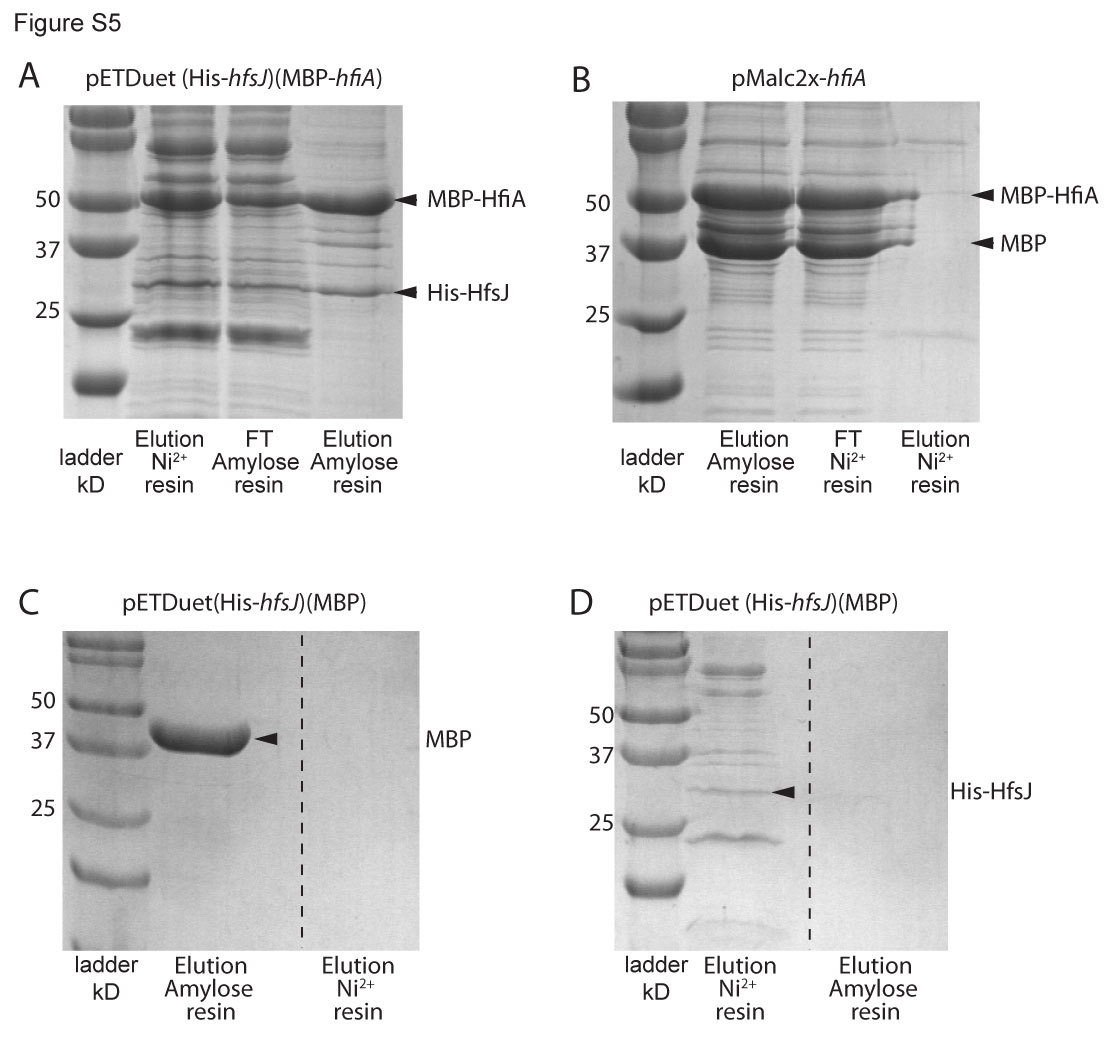

Supplement: Figure S5 — HfiA – HfsJ co-expression and co-purification controls. Plasmids used and proteins expressed are indicated above each SDS-PAGE gel. Aliquots of purified proteins are loaded left to right reflecting sequential purification steps. Relevant bands are indicated by black arrowheads. A. Co-expression and purification similar to that described in the main text, but in the reverse order, starting with Ni2+ resin to capture His6-HfsJ followed by amylose resin to capture MBP-HfiA. Increased non-specific binding to Ni2+ resin in step 1 results in reduced final purity of isolated proteins. Nevertheless, co-purification is observed after both Ni2+ and amylose resin purification steps. B. When MBP-HfiA is expressed from a pMal-c2x plasmid, MBP-HfiA along with a protein the size of MBP lacking the HfiA fusion elute from amylose resin as expected. However, neither of these species bind and elute from Ni2+ resin indicating that co-purification is not mediated by an interaction between MBP or HfiA with the Ni2+ resin. C. MBP without the HfiA fusion does not co-purify with co-expressed His6-HfsJ. His6-HfsJ is not detected after affinity purification using amylose resin or enriched in a second round of purification with Ni2+ resin. D. MBP without the HfiA fusion does not co-purify with His6-HfsJ. Together the results in B, C and D indicate that co-purification of His6-HfsJ and MBP-HfiA is mediated by an interaction between HfsJ and HfiA, and not by spurious interactions with MBP or the purification resins. (JPG) [file pgen.1004101.s005.jpg]

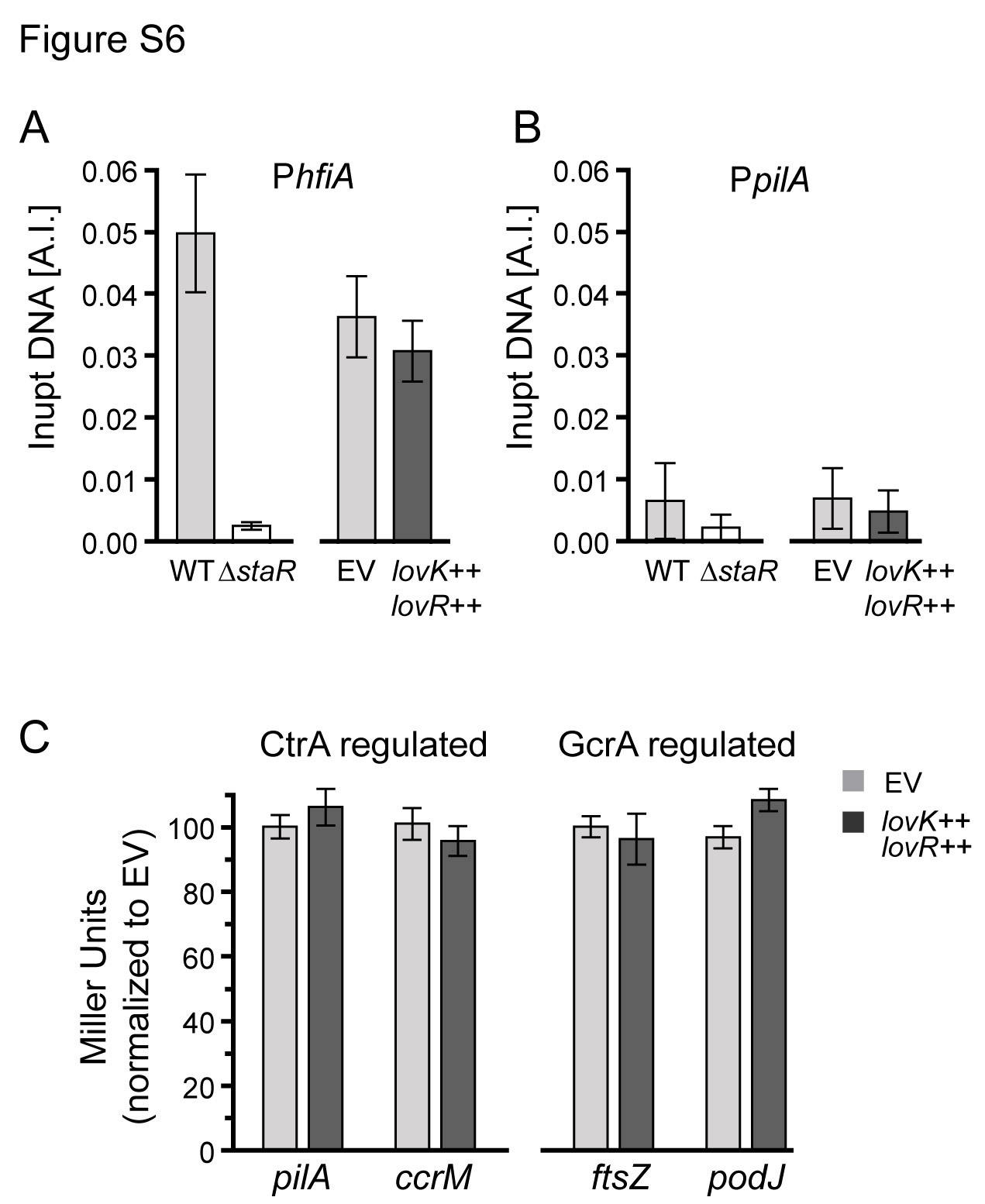

Supplement: Figure S6 — StaR, CtrA and GcrA promoter binding and transcriptional regulatory activities are not affected by lovK-lovR overexpression. A–B. Overexpression of lovK and lovR does not influence the efficiency of StaR precipitation of PhfiA. StaR-ChIP followed by qPCR on DNA precipitated from WT, ΔstaR, lovK-lovR overexpression or vector control (EV) strains. Primers amplified the hfiA promoter region (A) or the pilA promoter region (B) as a negative control region that is not occupied by StaR. Real-time PCR was performed using a Step-One Real-Time PCR system (Applied Biosystems, Foster City, CA) using 5 µL of each ChIP sample in a reaction with SYBR green PCR master mix (Quanta Biosciences, Gaithersburg, MD). Standard curve generated from the cycle threshold (Ct) value of the serially diluted chromatin input was used to calculate the percentage input value of each sample. Average values are from triplicate measurements done per culture. The final data were generated from three independent cultures. The DNA regions analyzed by real-time PCR were from nucleotide −147 to +126 relative to the start codon of hfiA and from −287 to −91 relative to the start codon of pilA with the following primers: hfiA ChIP F2- 5′AAACCACAACAACGAGGCCAA; hfiA ChIP R2- 5′ACGGACGTGATGCACTACAGCTA; pilA ChIP F- 5′CGACTGCACTTAATGGCCAG; and pilA ChIP R- 5′GCCAGCATCACTTTCTTTGG. C. β-galactosidase activity from transcriptional fusions between known CtrA or GcrA regulated promoters and lacZ was evaluated in strains overexpressing lovK and lovR (dark grey) and in empty vector (EV) control strains (light grey). Promoters assayed are indicated on the x-axis. No significant differences were observed upon lovK-lovR overexpression. (JPG) [file pgen.1004101.s006.jpg]

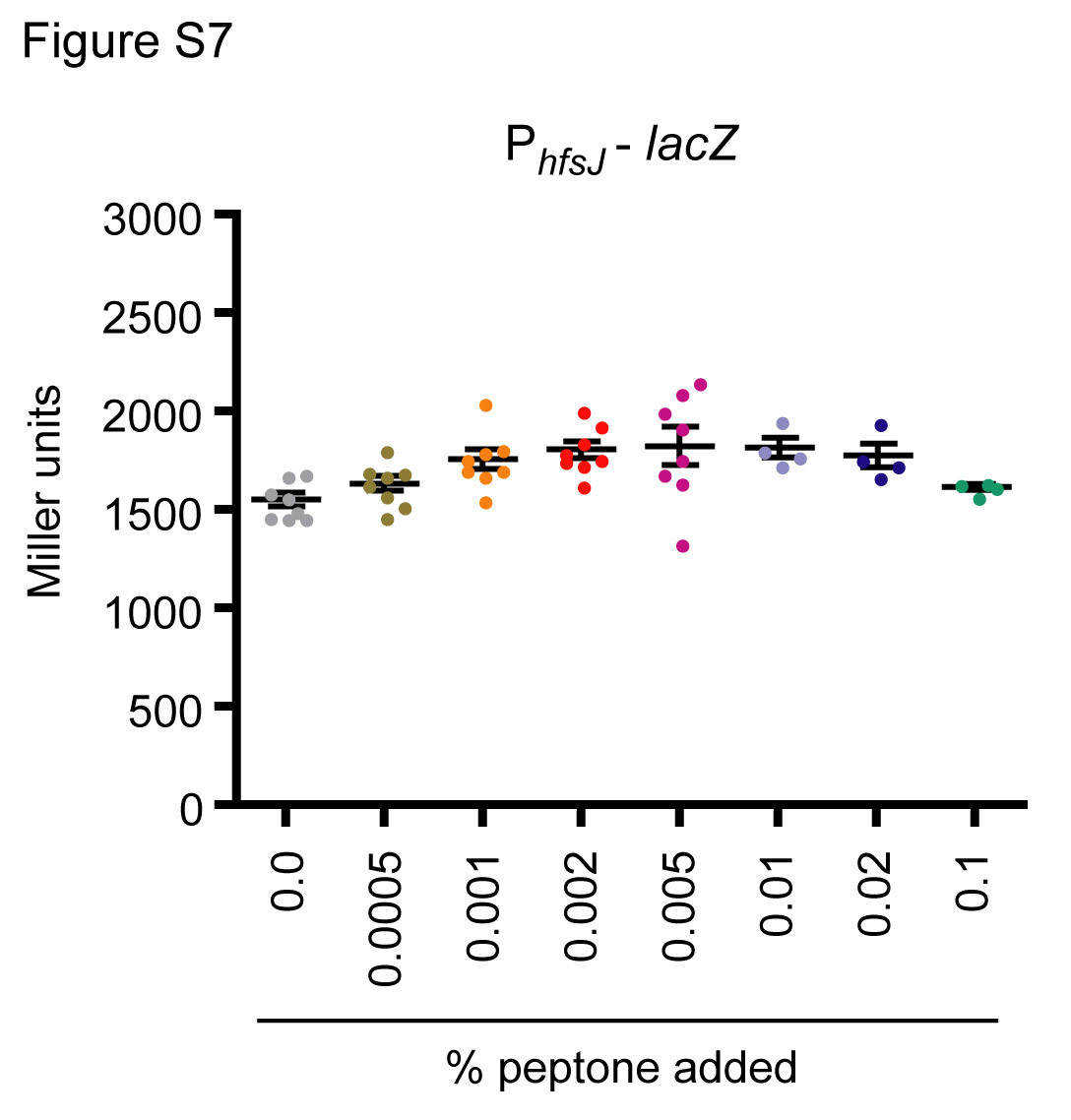

Supplement: Figure S7 — hfsJ transcription is not significantly affected by the nutrient content of the culture medium. β-galactosidase activity from the PhfsJ-lacZ transcriptional fusion (pRKlac290-PhfsJ) was measured in wild-type cells grown in M2X defined minimal medium supplemented with increasing amounts of peptone. As in Figure 6, starter cultures were diluted to a low OD so that after ∼16 hours of growth, the OD660 of the culture was ∼0.1. Dots represent individual measurements from independent cultures collected over 2 different days colored as in Figure 6. Black lines represent the mean ± s.e.m. Differences between conditions were statistically assessed with ANOVA followed by Tukey's multiple comparison post-test. No significant (p<0.05) differences were found. (JPG) [file pgen.1004101.s007.jpg]
