# Supplementary material for: A Cell Cycle and Nutritional Checkpoint Controlling Bacterial Surface Adhesion
Source: PLoS Genet. 2014 Jan 23;10(1):e1004101. doi: 10.1371/journal.pgen.1004101 (PMC3900383; doi:10.1371/journal.pgen.1004101)
Supplement: Table S1 — Spontaneous mutations that suppress the hfiA overexpression phenotype. (PDF) [file pgen.1004101.s008.pdf]

Table S1: Spontaneous mutations that suppress the *hfiA* overexpression phenotype.

*Group 1: Plasmid mutations in pMT805-*hfiA *identified by targeted sequencing of the plasmid*

**Type of change**	**Nucleotide change[1]**	**Coding change[2]**	**Notes[3]**
Nonsense SNP	C16T	R6stop	
Nonsense; 1 bp insert	TT(23-24)TTT	D9stop	Partially active
Nonsense SNP	C28T	Q10stop	Partially active
Nonsense SNP	C31T	R11stop	
2 non-synonymous SNPs	T68A, T71A	I23N, L24H	
Nonsense SNP	C73T	Q25stop	
Non-synonymous SNP	T101G	L34R	
Non-synonymous SNP	T104A	V35E	
In-frame 15 bp duplication	2X C109-G123	2X(L37-A42)	Dominant
Frameshift; 1 bp insert	CG(117-118)CAG	Adds 68 codons after P39	
Frameshift; 82 bp deletion	ΔG123-C214	Adds 30 codons after A40	
Nonsense SNP	T155A	L52stop	
Frameshift; 1 bp insert	C_6(204-209)C_7	Adds 41 codons after P66	

Group 2: Chromosomal mutations

**Mutant**	**Locus**	**Function**	**Type of change**	**Nucleotide change[4,5]**	**Coding change[5]**
Group 2a: Mutations mapped by whole genome sequencing					
256-39	**CC_0095**	UDP-glycosyl transferase	SNP	T778C	C260R
	CC_2868	NeuB, N-acetylneuraminate synthase	SNP	T3118669C	5' non-coding
261-15	**CC_0095**	UDP-glycosyl transferase	2 SNPs	T778C, T790C	C260R, W264R
	CC_2864	ArsR family transcription regulator	SNP	A194G	H65R
Group 2b: Mutations identified by targeted sequencing of only CC_0095 locus					
256-112	CC_0095	UDP-glycosyl transferase	SNP	T743G	L248R
256-177	CC_0095	UDP-glycosyl transferase	Deletion: 68 bp	ΔT800-G867	Frame shift after L266
256-185	CC_0095	UDP-glycosyl transferase	Duplication: 30 bp	2X T736-G765	2X F246-L255

[1]Nucleotide position is indicated with reference to the reannotated translation start site corresponding to position 938825 in the NA1000 reference genome (Genbank accession CP001340); see also Figure S3.

[2]Amino acid residues are indicated with reference to the reannotated HfiA primary sequence; see also Figure S3.

[3]*Partially active:* Surface binding, holdfast development and rosette formation not fully restored to WT / pMT805 (EV) levels in these mutants. *Dominant:* This mutation leads to enhanced surface binding, holdfast development and rosette formation compared to WT / pMT805 (EV) suggesting it may also affect endogenously expressed HfiA.

[4]Coding changes are in gene coordinates; coordinate of non-coding change corresponds to position in the NA1000 reference genome.

[5]CC_0095 coordinates correspond to the start codon annotated in the NA1000 reference genome (CCNA_0094)
