# Supplementary material for: A Cell Cycle and Nutritional Checkpoint Controlling Bacterial Surface Adhesion
Source: PLoS Genet. 2014 Jan 23;10(1):e1004101. doi: 10.1371/journal.pgen.1004101 (PMC3900383; doi:10.1371/journal.pgen.1004101)
Supplement: Table S6 — Plasmids, primers and strains used. (PDF) [file pgen.1004101.s013.pdf]

Table S6: Plasmids, primers and strains used

Strain #	Genotype	Comments / primers to amplify inserted sequence	Source
***E. coli* strains with plasmids**			
FC929	TOP10	Cloning strain	Invitrogen
FC3	MT607 / pRK600	Helper strain for tri-parental matings	(Finan et al., 1986)
FC803	Rosetta (DE3) pLysS	Strain for protein expression from T7 promoter	Novagen
***Caulobacter* expression plasmids**			
FC339	TOP10 / pMT585 (pXGFPC-2)	Integrates at the xylose locus, KanR	(Thanbichler et al., 2007)
FC338	TOP10 / pMT528 (pVCHYC-1)	Integrates at the vanillate locus, Spec/StrepR	(Thanbichler et al., 2007)
MTLS 4389	TOP10 / pMT680 (pXGFPC-6)	Integrates at the xylose locus, ChloR	(Thanbichler et al., 2007)
MTLS 4322	TOP10 / pMT581 (pXMCS-4)	Integrates at native locus, xylose-inducible promoter, GentR	(Thanbichler et al., 2007)
MTLS 4423	TOP10 / pMT805 (pBXMCS-6)	Replicating plasmid (mid-copy, BBR origin) with xylose-inducible promoter, ChlorR	(Thanbichler et al., 2007)
MTLS 4421	TOP10 / pMT464 (pBXMCS-2)	Replicating plasmid (mid-copy, BBR origin) with xylose-inducible promoter, KanR	(Thanbichler et al., 2007)
MTLS 4322	TOP10 / pMT666 (pVENC-2)	Integrates at native locus and generates a C-terminal fusion to venus, KanR	(Thanbichler et al., 2007)
MTLS 4424	TOP10 / pMT630 (pBVMCS-2)	Replicating plasmid (low copy, RK2 origin) with vanillate-inducible promoter, KanR	(Thanbichler et al., 2007)
FC364	TOP10 / pMT585-*lovR*		(Purcell et al., 2007)
FC432	TOP10 / pMT528-*lovK*		(Purcell et al., 2007)
FC1361	TOP10 / pMT680-*hfiA*	F: catatgTCGGGCCATTTATTGAG<br>R: ggtaccGTCGGTCGTGCGCATAGT	This work
pAF465	TOP10 / pMT581-*hfiA*	F: catatgTCGGGCCATTTATTGAG<br>R: ggtacctCTAGAGCGTTTGCGGTTC	This work
pAF463	TOP10 / pMT805-*hfiA*	F: catatgTCGGGCCATTTATTGAG<br>R: ggtaccGTCGGTCGTGCGCATAGT	This work
pAF493	TOP10 / pMT680-*staR*	F: aatacatatgGCTTCCATGGGCAAC<br>R: aataggtaccGTACGCGGTGGTCGACTTTA	This work
pAF498	TOP10 / pMT630-*hfsJ*	F: atatcatatgTTCGTTCCTCAAGAGC<br>R: aataggtaccCCTGAAGTTCAACAGCAT	This work
FC2016	TOP10 / pMT464-*wecG*$_{(E.c.)}$	F: atatcatatgAATAACAACACCACGGC<br>R: atatgaattcTCATAGGTTGCCGGTGTAGT	This work
FC2017	TOP10 / pMT464-*tagA*$_{(B.s.)}$	F: atatcatatgCAAACAGAGACTATTCACA<br>R: atatgaattcTTAAATCTGTTTTGTATGATCTTTTTC	This work
FC2018	TOP10 / pMT666-P$_{hfsJ}$-*hfsJ*-venus	F: atatcatatgTGACGCTCTTGAAGTCGTGCG<br>R: atatgaattcGAGCGCGCCGCCTCGTTG	This work
**Allele replacement plasmids**			
FC55	DH10B / pNPTS138	Allele replacement plasmid, KanR, SacB	M.R.K. Alley
FC1357	TOP10 / pNPTS138-Δ*hfiA*	UP F: ggatccGATGTGGCAGTTCCTGTTGA<br>UP R+:<br>AGCGGCGAAACCTCAATAAATGGCCCGACAC<br>DN F+: CATTTATTGAGGTTTCGCCGCTCGAAGAT<br>DN R: gaatTCCAGCTCGACGGATTG	This work
FC725	TOP10 / pNPTS138-Δ*hfsA*	UP F: gaattcCCGCCCACGAACACCTTCAA<br>UP R: ggatccGATCCAGGCGCTCGTCGACAT<br>DN F: ggatccCGGTGAGCGCTTTGGTCG<br>DN R: aagcttGCAGTCCACGACGATGACCT	This work
pAF491	TOP10 / pNPTS138-Δ*staR*	UP F: attactgcagATCACCTCCCAGTTCGACAG<br>UP R+: ACTTGGCCATGTCGTTGCCCATGGAAG<br>DN F+: CAACGACATGGCCAAGTCGCTGAACG<br>DN R: attagaattcCGATGAAGATCTCATTGACG	This work
pAF494	TOP10 / pNPTS138-Δ*hfsJ*	UP F: atatgaattcAAGGAACTGGCCAACCTGAT<br>UP R+: GACAGCCTCTGCTCTTGAGGAACGA<br>DN F+: GAGCAGAGGCTGTCCAGACGCTCTAC<br>DN R: atatctgcaGACCATCGTTTCTTCCT	This work

pAF456	TOP10 / pNPTS138-<br>*hfiA*(ΔcodonC)	UP F: ggatccGATGTGGCAGTTCCTGTTGA<br>Δc UP R+: TCGGCTCACTGACAAgATCCT<br>Δc DN F+: TTGAGCGCGGACAGGATcTTG<br>DN R: gaatTCCAGCTCGACGGATTG	This work
pAF457	TOP10 / pNPTS138-<br>*hfiA*(ΔcodonD)	UP F: ggatccGATGTGGCAGTTCCTGTTGA<br>Δd UP R+: GGCTCACTGAgAgCATCCTG<br>Δd DN F+: CGGACAGGATGcTcTCAGTG<br>DN R: gaatTCCAGCTCGACGGATTG	This work
pAF460	TOP10 / pNPTS138-<br>*hfiA*(ΔcodonE)	UP F: ggatccGATGTGGCAGTTCCTGTTGA<br>Δe UP R+: GGCTgACTGACAACATCCTG<br>Δe DN F+: CGGACAGGATGTTGTCAGTc<br>DN R: gaatTCCAGCTCGACGGATTG	This work
pAF458	TOP10 / pNPTS138-<br>*hfiA*(ΔcodonC,D)	UP F: ggatccGATGTGGCAGTTCCTGTTGA<br>Δc,d UP R+: GGCTCACTGAgAggATCCTG<br>Δc,d DN F+: CGGACAGGATccTcTCAGTG<br>DN R: gaatTCCAGCTCGACGGATTG	This work
pAF478	TOP10 / pNPTS138-<br>*hfiA*(ΔcodonC,E)	UP F: ggatccGATGTGGCAGTTCCTGTTGA<br>Δc,e UP R+: TCGGCTgACTGACAAgATCCT<br>Δc,e DN F+: TTGAGCGCGGACAGGATcTTG<br>DN R: gaatTCCAGCTCGACGGATTG	This work
pAF479	TOP10 / pNPTS138-<br>*hfiA*(ΔcodonD,E)	UP F: ggatccGATGTGGCAGTTCCTGTTGA<br>Δd,e UP R+: GGCTgACTGAgAgCATCCTG<br>Δd,e DN F+: CGGACAGGATGcTcTCAGTc<br>DN R: gaatTCCAGCTCGACGGATTG	This work
pAF489	TOP10 / pNPTS138-<br>*hfiA*(ΔcodonC,D,E)	UP F: ggatccGATGTGGCAGTTCCTGTTGA<br>Δc,d,e UP R+: GGCTgACTGAgTggATCCTG<br>Δc,d,e DN F+: CGGACAGGATccTcTCAGTc<br>DN R: gaatTCCAGCTCGACGGATTG	This work
FC2099	TOP10 / pNPTS138-<br>*hfsJ*(ΔcodonA)	UP F: attagaattcGATCGGCCAGTTCTACAAGG<br>ΔA UP R: CTCCGCAGAATaTCTCCAAG<br>ΔA DN F: CTTGGAGAtATTCTGCGGAG<br>DN R: ttttctgcaGACCATCGTTTCTTCCT	This work
FC2101	TOP10 / pNPTS138-<br>*hfsJ*(ΔcodonB)	UP F: attagaattcGATCGGCCAGTTCTACAAGG<br>ΔB UP R: CTGCGGAGATaTTCGTTCC<br>ΔB DN F: GGAACGAAtATCTCCGCAG<br>DN R: ttttctgcaGACCATCGTTTCTTCCT	This work
pAF495	TOP10 / pNPTS138-<br>*hfsJ(C260R)*	UP F: attagaattcGATCGGCCAGTTCTACAAGG<br>T778C UP R+: GATGCgGTAGCGGAAGAAC<br>T778C DN F+: GTTCTTCCGCTAC**c**GCATC<br>DN R: ttttctgcaGACCATCGTTTCTTCCT	This work
pAF497	TOP10 / pNPTS138-<br>*hfsJ(C260R, W264R)*	UP F: attagaattcGATCGGCCAGTTCTACAAGG<br>T778,790C UP R+: CgGGGCTCGATGCgGTAG<br>T778,790C DN F+: TAC**c**GCATCGAGCCC**c**GGT<br>DN R: ttttctgcaGACCATCGTTTCTTCCT	This work
pAF524	TOP10 / pNPTS138-<br>*hfsJ(L248R)*	UP F: attagaattcGATCGGCCAGTTCTACAAGG<br>DN R: ttttctgcaGACCATCGTTTCTTCCT<br>Amplified allele from sup256-112	
pAF525	TOP10 / pNPTS138-<br>*hfsJ(L266fs)*	UP F: attagaattcGATCGGCCAGTTCTACAAGG<br>DN R: ttttctgcaGACCATCGTTTCTTCCT<br>Amplified allele from sup256-177	
pAF526	TOP10 / pNPTS138-<br>*hfsJ(2XF246-L255)*	UP F: attagaattcGATCGGCCAGTTCTACAAGG<br>DN R: ttttctgcaGACCATCGTTTCTTCCT<br>Amplified allele from sup256-185	
**Transcription or translational *lacZ* fusion plasmids**			
FC54	S17-1 / pRKlac290	Plasmid for *lacZ* transcriptional fusions	(Gober and Shapiro, 1992)
pAF427	TOP10 / pRKlac290-P*hfiA*	F: ggtaccCTTCCTGCGCATCAACGAT<br>R: aagcttTCAATAAATGGCCCGACAC	This work
LT152	TOP10 / pRKlac290-P*hfsJ*	F:<br>aaaaaagaattcACCCCAAGGGCCACGCCCAGAA<br>R:<br>aaaaaatctagaGGCCCACCAGGCGCGCCCAGAA	This work
FC1101	TOP10 / pPR9TT	Plasmid for lacZ transcriptional and translational fusions	(Santos et al., 2001)
pAF429	TOP10 / pPR9TT-P*hfiA*(*a,b*)	ggtaccCTTCCTGCGCATCAACGAT<br>aagcttCTCAATAAATGGCCCGACAC	This work
pAF431	TOP10 / pPR9TT- P*hfiA*(*a-e)*	ggtaccCTTCCTGCGCATCAACGAT<br>aagctTCGGCTCACTGACAACATC	This work

**Heterologous protein expression plasmids**			
FC119	TOP10 / pMal-c2x	Plasmid to express proteins fused to maltose binding protein (MBP)	New England Biolabs
FC2001	TOP10 / pMal-c2x-*hfiA*	F: taatgaattcaTGTCGGGCCATTTATTGAG<br>R: taatctgcagGTCGGTCGTGCGCATAGT	This work
FC274	TOP10 / pETDuet	Plasmid for co-expression of proteins from two different T7 promoters	Novagen
FC2011	TOP10 / pETDuet(His-*hfsJ*)(MBP)	*hfsJ* amplified from genomic DNA<br>hfsJ F: taatgaattcCGTTCTGGGCGGGACCAT<br>hfsJ R: aatcaagcttCCCCTGAAGTTCAACAGCAT<br>MBP amplified from pMal-c2X-*hfiA*<br>MBP F: TTCACGAGCACTTCACCAAC (NdeI)<br>MBP R: atatggtaccTCATGAAATCCTTCCCTCGATCC	This work
FC2003	TOP10 / pETDuet(His-*hfsJ*)(MBP-*hfiA*)	*hfsJ* amplified from genomic DNA<br>hfsJ F: taatgaattcCGTTCTGGGCGGGACCAT<br>hfsJ R: aatcaagcttCCCCTGAAGTTCAACAGCAT<br>MBP-hfiA amplified from pMal-c2X-*hfiA*<br>MBP-hfiA-F: TTCACGAGCACTTCACCAAC (NdeI)<br>MBP-hfiA-R: aataggtaccGTGGGTCGTGCGCATAGT	This work
**Bacterial two hybrid strains and plasmids**			
FC257	BTH101 (F- cya-99, araD139, galE15, galK16, rpsL1 (Str resistant), hsdR2, mcrA1, mcrB1)	Adenylate cyclase null strain for bacterial two-hybid assay	(Karimova et al., 1998)
FC253	TOP10 / pUT18c	Parent plasmid to create fusions to the C-terminal end of adenylate cyclase T18	(Karimova et al., 1998)
FC255	TOP10 / pKT25	Parent plasmid to create fusions to the C-terminal end of adenylate cyclase T25	(Karimova et al., 1998)
FC254	TOP10 / pUT18c-zip	Control plasmid with the adenylate cyclase T18 domain fused to the leucine zipper of GCN4	(Karimova et al., 1998)
FC256	TOP10 / pKT25-zip	Control plasmid with the adenylate cyclase T25 domain fused to the leucine zipper of GCN4	(Karimova et al., 1998)
FC2006	TOP10 / pKT25-*hfsJ*	F: atattctagaGGTTCTGGGCGGGACCATG<br>R: atatggtaccTTAGCGCGCCGCCTCGTT	This work
FC2007	TOP10 / pKT25-*hfsJ(C260R)*	Same as above with pAF495 as template	This work
FC2009	TOP10 / pKT25-*hfsJ(C260R, W264R)*	Same as above with pAF497 as template	This work
FC2128	TOP10 / pKT25-*hfsJ(L248R)*	Same as above with pAF524 as template	This work
FC2129	TOP10 / pKT25-*hfsJ(L256fs)*	Same as above with pAF525 as template	This work
FC2130	TOP10 / pKT25-*hfsJ(2X(F246-L255))*	Same as above with pAF526 as template	This work
FC2010	TOP10 / pUT18c-*hfiA*	F: atatggatccGATGTCGGGCCATTTATTGAGC<br>R: atatgaattcTCAGCGGCGGGGGGC	This work

	***Caulobacter crescentus* strains**	**Source**	**Figure**
FC19	Wild type CB15	(Poindexter, 1964)	2, S2, S4, S6, 6
FC423	CB15 *xylX*:pMT585 *vanR*:pMT528	(Purcell et al., 2007)	1, S1, S6
FC438	CB15 *xylX*:pMT585-lovR *vanR*:pMT528-*lovK*	(Purcell et al., 2007)	1, S1, 2, S6
FC1289	CB15 Δ*hfsA xylX*::pMT585 *vanR*::pMT528	This work	1, S1
FC1290	CB15 Δ*hfsA xylX*::pMT585-*lovR vanR*::pMT528-*lovK*	This work	1, S1
FC1244	CB15 Δ*sigT xylX*::pMT585 *vanR*::pMT528	(Foreman et al., 2012)	1, S1
FC1247	CB15 Δ*sigT xylX*::pMT585-*lovR vanR*::pMT528-*lovK*	(Foreman et al., 2012)	1, S1
FC1949	CB15 / pRKlac290-P*hfiA*	This work	1, 5, 6
FC1950	CB15 *xylX*::pMT585 *vanR*::pMT528 / pRKlac290-P*hfiA*	This work	1
FC1951	CB15 *xylX*::pMT585-*lovR* vanR::pMT528-*lovK* / pRKlac290-P*hfiA*	This work	1
FC1398	CB15 *xylX*::pMT680	This work	2, 5
FC1399	CB15 *xylX*::pMT680-*hfiA*	This work	2
FC1365	CB15 Δ*hfiA*	This work	2, S2, 6
ACC349	CB15 Δ*hfiA xylX*::pMT680	This work	2

ACC272	CB15 Δ*hfiA xylX*::pMT680-*hfiA*	This work	2
FC1370	CB15 *xylX*::pMT585-*lovR vanR*::pMT528-*lovK xylX*::pMT680	This work	2
FC1371	CB15 *xylX*::pMT585-*lovR vanR*::pMT528-*lovK xylX*::pMT680-*hfiA*	This work	2
FC1986	CB15 / pPR9TT	This work	S2
FC1987	CB15 / pPR9TT- P*hfiA*(*a,b*)	This work	S2
FC1988	CB15 / pPR9TT- P*hfiA*(*a-e*)	This work	S2
FC1990	CB15 *hfiA*(ΔcodonC)	This work	S2
FC1991	CB15 *hfiA*(ΔcodonD)	This work	S2
FC1992	CB15 *hfiA*(ΔcodonE)	This work	S2
FC1993	CB15 *hfiA*(ΔcodonC,D)	This work	S2
FC1994	CB15 *hfiA*(ΔcodonC,E)	This work	S2
FC1995	CB15 *hfiA*(ΔcodonD,E)	This work	S2
FC1997	CB15 *hfiA*(ΔcodonC,D,E)	This work	S2
FC1934	CB15 / pMT805	This work	3
FC1935	CB15 / pMT805-*hfiA* (sup256 parent strain)	This work	3
FC1936	CB15 *hfiA*::pMT581-Pxyl-*hfiA xylX*::pMT680-*hfiA* (sup261 parent strain)	This work	3
FC1943	CB15 sup256-39	This work	3
FC1944	CB15 sup261-15	This work	3
FC1945	CB15 sup256-112	This work	Table S1
FC1946	CB15 sup256-177	This work	Table S1
FC1947	CB15 sup256-185	This work	Table S1
FC1974	CB15 Δ*hfsJ*	This work	3, S4
FC1975	CB15 *hfsJ*(C260R)	This work	
FC1977	CB15 *hfsJ*(C260R,W264R)	This work	
FC2119	CB15 *hfsJ*(L248R)	This work	
FC2120	CB15 *hfsJ*(L256fs))	This work	
FC2121	CB15 *hfsJ*(2X(F246-L255))	This work	
FC1978	CB15 *hfsJ*(C260R) / pMT805	This work	3
FC1981	CB15 *hfsJ*(C260R) / pMT805-*hfiA*	This work	3
FC1980	CB15 *hfsJ*(C260R,W264R) / pMT805	This work	3
FC1983	CB15 *hfsJ*(C260R,W264R) / pMT805-*hfiA*	This work	3
ACC392	CB15 *hfsJ*(L248R) / pMT805	This work	3
ACC393	CB15 *hfsJ*(L248R) / pMT805-*hfiA*	This work	3
ACC394	CB15 *hfsJ*(L256fs / pMT805	This work	3
ACC395	CB15 *hfsJ*(L256fs) / pMT805-*hfiA*	This work	3
ACC396	CB15 *hfsJ*(2XF246-L255) / pMT805	This work	3
ACC397	CB15 *hfsJ*(2XF246-L255) / pMT805-*hfiA*	This work	3
FC2100	CB15 *hfsJ*(ΔcodonA)	This work	S4
FC2102	CB15 *hfsJ*(ΔcodonB)	This work	S4
FC380	CB15 / pMT630	This work	S4
ACC371	CB15 Δ*hfsJ* / pMT630	This work	S4
ACC372	CB15 Δ*hfsJ* / pMT630-*hfsJ*	This work	S4
FC646	CB15 / pMT464	This work	S4
FC2090	CB15 ΔhfsJ / pMT464	This work	S4
FC2019	CB15 Δ*hfsJ* / pMT464-*wecG*	This work	S4
FC2020	CB15 Δ*hfsJ* / pMT464-*tagA*	This work	S4
FC2021	CB15 Δ*hfsJ*::pMT666-P_{hfsJ}-*hfsJ*-venus	This work	S4
FC1952	NA1000 / pRKlac290-P*hfiA*	This work	5
FC1953	NA1000 *ctrA(V88G)*ts / pRKlac290-P*hfiA*	Parent strain (Jacobs et al., 1999)	5
FC1954	NA1000 *ctrA(T170I)ts* / pRKlac290-P*hfiA*	Parent strain (Quon et al., 1996)	5
FC1955	NA1000 *gcrAΩ xyl*::*gcrA* / pRKlac290-P*hfiA*	Parent strain (Holtzendorff et al., 2004)	5
FC1961	CB15 *xylX*::pMT680 / pRKlac290-P*hfiA*	This work	5
FC1962	CB15 *xylX*::pMT680-*staR* / pRKlac290-P*hfiA*	This work	5
FC1960	CB15 Δ*staR* / pRKlac290-P*hfiA*	This work	5

FC1963	CB15 Δ*staR* *xylX*::pMT585 *vanR*::pMT528 / pRKlac290-P*hfiA*	This work	5
FC1964	CB15 Δ*staR* *xylX*::pMT585-lovR *vanR*::pMT528-*lovK* / pRKlac290-P*hfiA*	This work	5
LT396	NA1000 / pRKlac290-P*hfsJ*	This work	5
LT397	NA1000 *ctrA(T170I)ts* / pRKlac290-P*hfsJ*	Parent strain (Quon et al., 1996)	5
FC1972	CB15 *xylX*::pMT680-*staR*	This work	5
FC1970	CB15 Δ*staR*	This work	S6
FC2071	CB15 / pRKlac290-P*hfsJ*	This work	S7
