# Supplementary material for: A Cell Cycle and Nutritional Checkpoint Controlling Bacterial Surface Adhesion
Source: PLoS Genet. 2014 Jan 23;10(1):e1004101. doi: 10.1371/journal.pgen.1004101 (PMC3900383; doi:10.1371/journal.pgen.1004101)
Supplement: Text S1 — Supplemental experimental procedures. (PDF) [file pgen.1004101.s014.pdf]

Text S1: Supplemental Experimental Procedures

General growth conditions

Escherichia coli strains were cultured in LB liquid medium or grown on LB-agar (15 g/L) at 37° C supplemented with antibiotics as appropriate at the following concentrations: kanamycin 50 μg/ml, tetracycline 12 μg/ml and spectinomycin/streptomycin 50 μg/ml / 30μg/ml, chloramphenicol 20 μg/ml, ampicillin 100 μg/ml.

Caulobacter crescentus colonies were grown on solid PYE medium (0.2% peptone, 0.1% yeast extract, 1.5% agar) [1] at 30° C. Liquid *C. crescentus* cultures were grown in PYE (0.2% peptone, 0.1% yeast extract, 1 mM $MgSO_4$, 0.5 mM $CaCl_2$) or in M2 defined medium [1] supplemented with 0.15% xylose (M2X) as the carbon source. To induce expression from P_{van}, 500 μM vanillate (final concentration) was added to all cultures in experiment that included P_{van}-*lovK* bearing strains (M2VX medium). Antibiotics were used at the following concentrations: kanamycin 5 μg/ml liquid, 25 μg/ml solid; tetracycline 1 μg/ml liquid, 2 μg/ml solid; and chloramphenicol 0.75 μg/ml for strains with integrated, single copy plasmids, 1.5 μg/ml for strains with replicating, multi-copy plasmids.

Plasmid construction

C. crescentus, E. coli or *B. subtilis* DNA was amplified using KOD Xtreme hot-start polymerase (EMD Millipore) and supplementing reactions with 5% DMSO. Restriction sites for cloning were added to the ends of the primers. Amplified products were digested with appropriate restriction enzymes (New England Biolabs) and ligated into similarly digested, phosphatase-treated and gel-purified plasmids using T4 DNA ligase (New England Biolabs). Plasmid ligations were transformed into *E. coli* Top10 (Life Technologies, Invitrogen). All cloned products were sequence confirmed.

Point mutant and null alleles were generated using an overlap extension PCR strategy [2] and unique restriction enzyme cut sites in the outermost primer sequences. Amplified alleles were ligated into the corresponding restriction sites in pNPTS138.

Plasmids used and generated in this work are listed in Table S6. Primer sequences used to generate sequences to insert into generated plasmids are also listed in Table S6.

Strain construction

Allele replacement and in-frame deletion strains were constructed using a double recombination strategy [3]. Each pNPTS138-derived allele-replacement plasmid was transformed into *C. crescentus* by electroporation. Primary integrants were selected on PYE-Kan plates. Overnight growth in non-selective liquid medium followed by growth on solid PYE supplemented with 3% sucrose allowed identification of clones in which the plasmid had been excised in a second recombination event. The locus of interest was PCR amplified from kanamycin-sensitive clones. Deletion alleles were confirmed by PCR product size; point mutant alleles were confirmed by sequencing the PCR products.

pRKlac290-based transcriptional reporter plasmids were conjugated into *C. crescentus* strains by tri-parental mating [1] using the *E. coli* helper strain FC3 (see Table S6 for strains). Briefly, tri-parental matings were performed by mixing the donor *E. coli* strain, the helper strain, and the *C. crescentus* recipient strain in a 1:1:5 ratio. Mixed cells were incubated non-selectively on solid PYE for 12-24 hours. *C. crescentus* cells containing the desired plasmid were then selected on solid PYE containing nalidixic acid (20 μg/ml) to counterselect against *E. coli* and a plasmid-appropriate antibiotic.

All other plasmids were purified from *E. coli* TOP10 and electroporated into *C. crescentus* strains. Electroporations were performed using a BioRad MicroPulser following manufacturers settings for *E. coli*.

Plasmids for heterologous protein expression and purification were transformed into Rosetta 2 (DE3) pLysS (EMD Millipore, Novagen) by electroporation. Plasmids for the bacterial two-hybrid assay were co-transformed into an adenylate cyclase null strain of *E. coli*, BTH101 [4], by electroporation.

Strains used and generated in this work are listed in Table S6.

Analysis of ChIP-seq sequences

The standard genomic position format files (BAM) were imported into SeqMonk (Braham http://www.bioinformatics.babraham.ac.uk/projects/seqmonk/, version 0.21.0) to build sequence read profiles. The initial quantification of the sequencing data was done in SeqMonk to allow the comparison of different conditions and to filter out region of interest. To this end, the genome was subdivided into 50 bp probes, and for every probe an associated value was calculated, a value that derives from the pattern of reads which occurs within the probe region used for the quantitation using the Red Count Quantitation option. To discern between background signal (modeled with a Poisson or negative binomial distribution) and candidate peaks, we calculated the ratio of reads per probe as a function of the total number of reads. The overall average read count (for all probes) plus twice the standard deviation was used to establish the lower cut-off that separates the background from candidate peaks. In most cases this cut-off was applied and it usually lay somewhere between 5-10 fold enrichment. To reduce the background, we modeled the reads distribution by a regression analysis of the reads count data, implemented in SPSS statistics (IBM Inc.) or R software (http://www.r-project.org/). The read count data were used to fit known distributions, and we set the cut off to a value which picked out the peaks from the background noise. Usually this was done by selecting the first twenty thousand probes and using the reads count data to fit known distributions. The best fit is used to calculate the tangent to the function that represents the distribution in the point x_0= -1. This point establishes the value at which to select the peaks from the background noise. Using this approach we were able to select only few peaks that represent a small portion of the dataset. In this case we used this selection in order to identify a value in between the lower cut off and tangent point in order to have a good compromise between the background noise signal and the candidates peaks.

Whole Genome Sequence analysis

Raw reads were processed by Cutadapt v1.1 to remove 3' adaptors and colors with quality score < 20 [5]. Alignment of clean reads to *C. crescentus CB15* reference genome (GI:19172958) was carried out by BFAST aligner v0.7.0a with settings "-A=1 –a=3", which maps reads at color-space and outputs only reads that are uniquely mapped to the genome with the best alignment score [6,7]. Duplicates were then removed by MarkDuplicates program from Picard tools v1.62 (http://picard.sourceforge.net). Genome coverage was calculated by DepthOfCoverage from Genome Analysis Toolkit (GATK) v1.4 [8]. Approximately 85% of reads from each sample mapped to the reference genome. Each sample had 45-50X average read coverage and 95% of bases were covered by at least 4 high-quality reads. For InDel/SNP calling, only reads with mapping quality score ≥ 30 and bases with base quality score ≥ 30 were used to identify the variants. InDels/SNPs in each sample were called simultaneously on all samples by SAMtools mpileup, filtered by VarScan v2.2.11 to output variants with ≥ 60% allele frequency and annotated by ANNOVAR [9-11]. Sample-unique variants with ≥ 95% allele frequency were identified by custom perl scripts and inspected by eye.

References

1. Ely B (1991) Genetics of *Caulobacter crescentus*. Methods Enzymol 204: 372-384.
2. Ho SN, Hunt HD, Horton RM, Pullen JK, Pease LR (1989) Site-directed mutagenesis by overlap extension using the polymerase chain reaction. Gene 77: 51-59.
3. Ried JL, Collmer A (1987) An *nptI-sacB-sacR* cartridge for constructing directed, unmarked mutations in gram-negative bacteria by marker exchange-eviction mutagenesis. Gene 57: 239-246.
4. Karimova G, Pidoux J, Ullmann A, Ladant D (1998) A bacterial two-hybrid system based on a reconstituted signal transduction pathway. Proc Natl Acad Sci U S A 95: 5752-5756.
5. Martin M (2011) Cutadapt removes adapter sequences from high-throughput sequencing reads. EMBnetjournal, North America 17.
6. Homer N, Merriman B, Nelson SF (2009) BFAST: an alignment tool for large scale genome resequencing. PLoS One 4: e7767.
7. Homer N, Merriman B, Nelson SF (2009) Local alignment of two-base encoded DNA sequence. BMC Bioinformatics 10: 175.
8. McKenna A, Hanna M, Banks E, Sivachenko A, Cibulskis K, et al. (2010) The Genome Analysis Toolkit: a MapReduce framework for analyzing next-generation DNA sequencing data. Genome Res 20: 1297-1303.
9. Wang K, Li M, Hakonarson H (2010) ANNOVAR: functional annotation of genetic variants from high-throughput sequencing data. Nucleic Acids Res 38: e164.
10. Koboldt DC, Zhang Q, Larson DE, Shen D, McLellan MD, et al. (2012) VarScan 2: somatic mutation and copy number alteration discovery in cancer by exome sequencing. Genome Res 22: 568-576.
11. Li H, Handsaker B, Wysoker A, Fennell T, Ruan J, et al. (2009) The Sequence Alignment/Map format and SAMtools. Bioinformatics 25: 2078-2079.
